# Revealing the reversible solid-state electrochemistry of lithium-containing conjugated oximates for organic batteries

**DOI:** 10.1126/sciadv.adg6079

**Published:** 2023-04-28

**Authors:** Jiande Wang, Petru Apostol, Darsi Rambabu, Xiaolong Guo, Xuelian Liu, Koen Robeyns, Mengyuan Du, Yan Zhang, Shubhadeep Pal, Robert Markowski, Fabio Lucaccioni, Alae Eddine Lakraychi, Cristian Morari, Jean-François Gohy, Deepak Gupta, Alexandru Vlad

**Affiliations:** ^1^ Institute of Condensed Matter and Nanosciences, Molecular Chemistry, Materials and Catalysis, Université catholique de Louvain, Louvain-la-Neuve B-1348, Belgium.; ^2^ College of Materials Science and Engineering, Hunan Province Key Laboratory for Advanced Carbon Materials and Applied Technology, Hunan University, Changsha 410082, Hunan, P. R. China.; ^3^ Institutul Național de Cercetare-Dezvoltare pentru Tehnologii Izotopice și Moleculare Cluj-Napoca, Cluj-Napoca, România.

## Abstract

In the rising advent of organic Li-ion positive electrode materials with increased energy content, chemistries with high redox potential and intrinsic oxidation stability remain a challenge. Here, we report the solid-phase reversible electrochemistry of the oximate organic redox functionality. The disclosed oximate chemistries, including cyclic, acyclic, aliphatic, and tetra-functional stereotypes, uncover the complex interplay between the molecular structure and the electroactivity. Among the exotic features, the most appealing one is the reversible electrochemical polymerization accompanying the charge storage process in solid phase, through intermolecular azodioxy bond coupling. The best-performing oximate delivers a high reversible capacity of 350 mAh g^−1^ at an average potential of 3.0 versus Li^+^/Li^0^, attaining 1 kWh kg^−1^ specific energy content at the material level metric. This work ascertains a strong link between electrochemistry, organic chemistry, and battery science by emphasizing on how different phases, mechanisms, and performances can be accessed using a single chemical functionality.

## Teaser

Conjugated oximates are organic Li-ion battery materials with high voltage and high energy content.

## INTRODUCTION

Organic electrode materials are considered as one potential option for future electrochemical energy storage applications owing to appealing features of intrinsic natural abundance of the constituent elements, sustainability, and low environmental footprint (*1*–*4*). The current battery value chain is exclusively dependent on transition metal electrode materials, which are procured after extensive mining and expensive synthesis protocols via energy consuming high-temperature processing. Material costs, geopolitical issues related to sourcing of these, and handling and recycling encourage the efforts toward development of alternatives, among which organic electrode materials fulfill many conditions. The recent fast advancement of organic electrodes indicates that these may not only emerge as mere alternatives to the traditional transition metal positive electrode materials in conventional rechargeable batteries but rather have the potential to lead to disruptive technologies (*5*).

The charge storage mechanism of organic positive electrode materials can be divided into “*n*-type” or “*p*-type” redox systems (*6*, *7*). While the former have been studied mainly in their oxidized state (requiring battery discharge at first utilization, thus being suitable only for the still underdeveloped lithium metal batteries), the latter stores the anion species, for application in dual-ion or anionic batteries (*8*). Conventional Li-ion cell assembly relies on a Li-ion source positive electrode material (the cathode) coupled to a Li-ion host material [the anode, in a rocking chair system (*9*, *10*)]. Compared to the large diversity of inorganic Li-ion positive materials, the foundation of the state-of-the-art practical organic Li-ion positive electrode materials (mostly developed over the past 4 years) was laid extensively on enolate-carbonyl redox chemistry. Encouraging advancements have been attained through electron-withdrawing substituted quinones (*11*), sacrificial metal-mediated charge delocalization (*12*, *13*), stereoelectronic chameleonic effect (*14*), and polymorphism Li_4_-*o*-DHT (β-phase) (*15*). Recent advances have led to the development of a new class of organic redox systems for batteries, the conjugated sulfonamide chemistry with intrinsic ambient air stability while in the Li-reservoir form (*16*). Whereas long considered challenging, the rationale of conjugated sulfonamides was based on decreasing the nucleophilicity of the organic anionic center, endowing resistance to hydrolysis and oxidation by molecular oxygen, while still allowing a reversible redox at a high potential. Unlocking the potential of this class of material not only provided new avenues for molecular engineering toward improved charge storage performances of also other monovalent cations but also encouraged the idea of exploring other organic redox systems with alike properties (*17*, *18*).

In this work, we present the application of conjugated oximate Lithium salts as positive electrode materials for batteries. We analyze and discuss the rich physicochemistry processes accompanying the electrochemistry in solid phase of these materials. To establish the versatility of the oximate redox chemistry as electrode materials for Li-ion batteries, five lithiated oximate systems with different chemical structures, including cyclic (aromatic), acyclic (nonaromatic), aliphatic, and tetra-functional stereotypes, are investigated. Each system is found to evolve differently upon redox, with characteristic features originating from specific intermolecular and intramolecular interactions. Among the peculiar characteristics found, the most intriguing include the solid-to-liquid reversible conversion of dilithium dimethylglyoxime upon redox and the reversible electrochemical polymerization, depolymerization accompanying the charge storage process in solid phase of the dilithium *p*-benzoquinone dioxime. The reversibility of the intermolecular azodioxy coupling (─ONNO─) or the intramolecular furoxan cyclization is also analyzed. The best-performing candidate (dilithium *p*-benzoquinone dioxime) displays a high reversible capacity of 350 mAh g^−1^ at a redox potential of 3 V versus Li^+^/Li^o^, reaching a specific energy density of nearly 1 kWh Kg^−1^, estimated at the material level. The excellent chemical and structural reversibility and the promising electrochemical performances of the studied conjugated oximates corroborate the potential of this class of materials as positive electrode materials for Li-ion batteries. The oximate redox functionality not only enriches the family of organic electrode materials (table S5) but also provides excellent redox reversibility, low molecular weight (equivalent of high capacity), and dry air stability of the anionic form, fulfilling the requirement of positive electrodes as candidates for Li-ion batteries.

## RESULTS AND DISCUSSION

### Design rationale and redox mechanisms of conjugated oximates

**Fig. 1.**
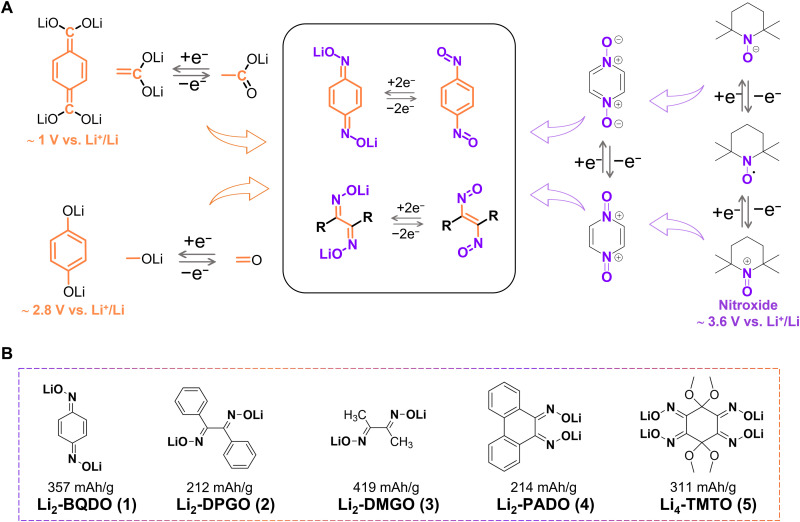
Design rationale and the library of conjugated oximates. (**A**) Left: Conjugated carboxylate and keto-enolate redox. Right: Nitroxide-based redox (oxoammonium cation ↔ nitroxide ↔ aminoxy anion). Molecular design of conjugated oximates (center) can be regarded as intramolecular hybrid fusion of di-aminoxy (right) and quinoneimine functions (left). (**B**) The first generation of conjugated oximate cathode materials disclosed in this work and the respective theoretical capacities and abbreviations used.

The skeleton of oximate redox functionality exemplifies the fusion of two important families of organic electrochemical storage mechanisms, namely, the conjugated carbonyls with the nitroxides (Fig. 1A). The conjugated carbonyl system (*n*-type) remains probably the most investigated and dominated the organic battery field with more than 100 structural analogs reported to date (*19*). The nitroxide redox (*p*-type), initially reported by Nishide, Oyaizu, and coworkers (*20*, *21*), has attracted rapid attention in the community given the high redox potential (3.6 V versus Li^+^/Li, *p*-type, anion storage) and the large versatility in incorporating this redox center into polymeric or small-molecular systems (*22*, *23*).

The reduced form of quinones (enolates) takes advantage of π-conjugation (aromatic system) to achieve stabilization through the mesomeric effect (*11*, *14*, *24*), whereas the nitroxide radical is an ambipolar redox center, with the nitroxide-oxoammonium *p*-type redox couple (3.6 V versus Li^+^/Li) being well studied, whereas the nitroxide-aminoxyl anion *n*-type redox couple (2.8 to 3.0 V versus Li^+^/Li) remaining elusive and less understood (*7*, *25*, *26*). Thus, the amalgamation of the concepts of charge stabilization by conjugation with the nitroxide-based localized redox would potentially result in a nitroxide-bearing conjugated π-system (e.g., pyrazine-*N*,*N*′-dioxide) that might favor radical stability and redox kinetics, although such a molecular system remains elusive so far (Fig. 1A, right hand).

On the other hand, in conjugated dicarboxylate systems, the redox potential of the carbonyl group is notably lower (in the range of 1 V versus Li^+^/Li, as compared to 2.0 to 3.0 V versus Li^+^/Li found in most carbonyl derivatives) owing to the strong electron donating effect of the ─OLi attached to the C═O carbon (the resonance carboxylate group), making these suitable as negative electrode materials for Li-ion storage (Fig. 1A, left) (*22*). We envisioned that the rationale of eschewing electron-donating group (─OLi) is a prerequisite for augmenting the redox potential, which would be possible by replacing the tetravalent carbon unit with a trivalent nitrogen atom, leading to the ketoximate group (═N─O^−^) that would merge the essential features of both nitroxide and carbonyl redox moieties. The benzoquinone dioximate [Fig. 1B (**1**), BQDO^2−^] thus represents a hybrid of iminoxy group and quinoneimine center with a reversible two-electron redox process, which was also found to be accompanied by a series of exotic processes, depending on the position of redox functionality and conjugation as discussed in the following sections. As the conjugated nonaromatic redox systems remain scarcely studied for organic battery electrode materials, a series of additional oximate derivatives [e.g., lithium salts of DPGO^2−^ (**2**), DMGO^2−^ (**3**), PADO^2−^ (**4**), and TMTO^4−^ (**5**), displayed in Fig. 1B] drew our attention as model compounds, due to their rich chemistry and low molecular weight, translating to design diversity and potentially high theoretical capacities (Fig. 1B).

All lithium salt of studied oximate anions (**1**–**5**) were prepared by direct acid-base reaction on the protonated versions at room temperature (refer to section "Materials Synthesis and Characterization" in the Supplementary Materials). Complete deprotonation was qualitatively confirmed by the disappearance of the 3200-cm^−1^ band of the hydroxyl group in Fourier transform infrared (FTIR) analysis (figs. S14 to S18). Being aware of the fact that air stability of cathode materials is a crucial parameter for practical applications and only a handful of organic positive Li-ion electrode materials comply to this requirement thus far (*11*, *12*, *14*, *16*), we focused our initial attention to the reactivity of the lithiated phases in ambient and controlled atmospheric environment. All the studied oximate (**1–5**) materials displayed no substantial chemical changes (asserted by FTIR analysis) after 48 hours of exposure to dry air, confirming the excellent oxidation stability of the oximate lithium salts (refer to figs. S14 to S18 and associated text for details on measurements and analysis).

The redox chemistry of oximes is relatively well studied, with primary interest on the anodically generated nitroso form (─N═O) that has industrial as well as fundamental considerations (*27*–*30*). Most of the organic nitroso compounds have been found to behave differently in dissolved and solid phases (for example, displaying pale yellow coloration in the solid state, as compared to bright green in solution) (*31*). This was attributed to the reversible dimerization of the nitroso compounds, existing as monomers in solution, and dimeric in the solid state, at room temperature. The (di)nitroso intermediates are stable only at low temperatures (<130 K) or elevated temperature in gas phase, with ability to dimerize or polymerize spontaneously at room temperature (*32*). The reversible dimerization of nitroso derivatives leads to the formation cis or trans azodioxides, or furoxan derivatives, depending on the electronic conjugation and molecular structure. The activation energy for the dissociation of azodioxy dimers is in the range of 20 to 30 kcal mol^−1^, suggesting that this covalent bond is strong but can nevertheless be broken and reformed reversibly under mild conditions. This aspect has been for example ingeniously exploited for the construction of monocrystalline covalent organic networks with a variety of linkers and topologies (*27*).

**Fig. 2.**
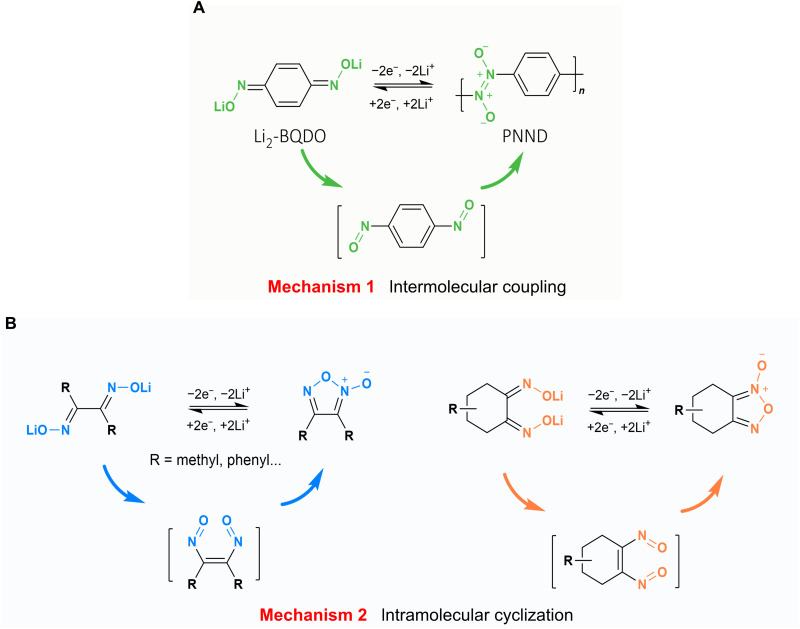
Reversible intra- and intermolecular conversion during redox in lithium oximates. (**A**) Redox mechanism of Li_2_-BQDO: Upon oxidation, Li_2_-BQDO exchanges two electrons and lithium ions forming the intermediate para-dinitrosobenzene, which undergoes intermolecular coupling to form PNND through azodioxy (─ONNO─) linkage. The corresponding abbreviations are shown below the molecules. (**B**) Left: Redox mechanism of 1,2-oximates (Li_2_-DPGO and Li_2_-DMGO): After oxidation, the dinitroso intermediate forms the furoxan ring via intramolecular cyclization. Right: Redox mechanism of ortho-oximates (α-dioximates) showing the formation of furoxan derivatives as the oxidation products.

However, despite the advances in understanding and application of nitroso-azidioxy chemistry, some fundamental differences and open questions and contradictions remain. Most studies have been performed so far on the dissolved phase of the protonated oximate forms, with sole emphasis on the chemical oxidation process. While the reversibility of the nitroso, azidoxy polymerization, depolymerization process is relatively well understood, the reversibility of the redox processes (either by chemical or electrochemical means) remains seldomly addressed and contradictory. This is an essential aspect of these materials to investigate, given the complex intra- and intermolecular conversion taking place (Fig. 2, and discussed further), that could be intuitively treated as irreversible. For example, some quinone dioximes were reported as irreversible, with only dimethyl ether derivatives showing reversible redox, yet proceeding through a *p*-type mechanism (formation of radical cation upon oxidation) (*29*). Our findings not only confirm the reversibility of the redox but also the reversibility of the subsequent dimerization-polymerization processes in the solid phase, beginning with either anionic (reduced) or the oxidized forms (cation-electron coupled events). All disclosed oximate lithium salts (**1–5**) were found to undergo a reversible one electron per oximate group redox, forming the intermediate nitroso derivatives, followed by reversible structural modifications, which are strongly dependent on electronic conjugation and molecular structure (Fig. 2). For instance, the electrochemical oxidation product of Li_2_-BQDO was identified as poly(1,4-phenyleneazine-*E*-*N*,*N*-dioxide) (PNND; mechanism 1), similar to the one obtained by chemical oxidation of *p*-benzoquinone dioxime with an excess of sodium hypochlorite in alkaline aqueous medium (*32*, *33*).

The in situ formation of PNND upon oxidation of Li_2_-BQDO was also confirmed by Rietveld refinement of the experimental powder x-ray diffraction (PXRD) data (figs. S19 and S20) (*34*). Only the product with *E*-(trans-)configuration around the O─N═N─O groups was detected, as also confirmed by the absence of the characteristic FTIR band for *Z*-(cis) conformer in the range of 1350 to 1400-cm^−1^ (*35*, *36*). This is consistent with the previous study on kinetics and thermodynamic stability of the PNND de/polymerization process, with exclusive formation of the *E-*configuration at ambient temperatures (*28*). The sequence of redox and de-polymerization steps in solid phase was found to be highly reversible, while being also exploited and proving advantageous for lithium storage. For instance, the PNND polymeric form was found to display low solubility in battery electrolytes (table S6 and fig. S21), the soluble species being composed of monomer, or low-unit oligomers (and not the polymeric form), as a result of azodioxy-nitroso equilibrium (*27*) (refer to the Supplementary Materials for additional details and discussion). The low solubility is an interesting feature for energy storage applications, given the general problem of elution of organic electrode materials typically arising from the oxidized/neutral phase (e.g., benzoquinone), unlike the PNND, which is poorly soluble in commercial electrolytes (table S6 and fig. S21) (*37*).

The α- or 1,2-dioximates (e.g., Li_2_-DPGO, Li_2_-PADO, and Li_4_-TMTO) also oxidize into the intermediate dinitroso states, which undergo intramolecular cyclization to furoxan derivatives as the final product [Fig. 2; mechanism 2, e.g., 3,4-diphenyl-1,2,5-oxadiazole-2-oxide (DPODO) as the oxidized phase of Li_2_-DPGO] (*32*, *38*, *39*). The redox of the linear aliphatic Li_2_-DMGO (**3**) proceeds through an additional unconventional solid-liquid conversion process, in that the oxidation reaction product (3,4-dimethyl furoxan) is liquid at ambient temperature, a feature that is highly suitable for redox flow battery systems (*40*, *41*). Despite the liquid phase of the end of charged product, the cell based on Li_2_-DMGO displayed high material utilization and good electrochemical reversibility at the first charge-discharge cycle (fig. S27). The formation of furoxan ring in solid state upon charge-discharge influences the redox process and changes the electrochemical performance of the electrode materials, which will be discussed in the next section.

### Solid-phase electrochemical and charge storage characteristics

**Fig. 3.**
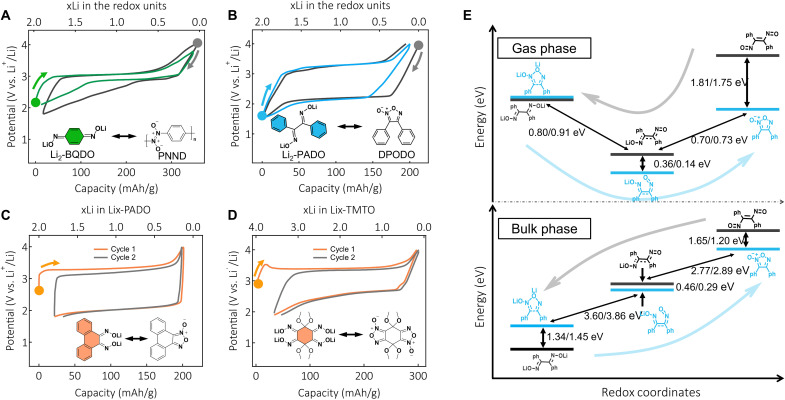
Solid-state electrochemistry of lithium oximates (1, 2, 4, and 5) as positive electrode material for charge storage. Potential-composition galvanostatic charge-discharge profiles of (**A**) Li_2_-BQDO (green curve) and PNND (gray curve) electrode materials cycled at a rate of C/10 (equivalent of 1e^−^/1Li^+^ exchanged in 5 hours), (**B**) Li_2_-DPGO (blue curve) and DPODO cells (black curve) electrode material, (**C**) Li_2_-PADO electrode material, and (**D**) of Li_4_-TMTO electrode material. (**E**) Redox coordinates for the conversion of Li_2_-DPGO to DPODO, considering reaction pathways for the linear and cyclic configurations, in both gas and solid phase (refer to "DFT calculation" section in the Supplementary Materials). The energy differences for phase transition (eV) are indicated for both experimental and calculated cell parameters.

Having established the structural and electrochemical features of the oximate-[nitroso]-azodioxy such as reversible electrochemical and chemical conversion, the solid-state electrochemical performance of **1–5** as positive electrode materials was evaluated next (Fig. 3). All studied compounds display the theoretical one-electron redox per oximate group, with average redox potential spanning the range of 2.5 to 3.1 V versus Li^+^/Li and efficient material utilization with high capacities of 210 to 350 mAh/g attained. For instance, the Li_2_-BQDO delivered 97% of the theoretical capacity (357 mAh g^−1^) at a potential around 3.0 V versus Li^+^/Li on the first cycle, corresponding to 1.94 Li^+^/e^−^ equivalents utilization (Fig. 3A, green curve, galvanostatic charge/discharge rate of one Li^+^ ion in 5 hours). The cells also displayed good rate capability (fig. S22), as well as stable behavior with more than 200 mAh g^−1^ retained after 50 cycles (over 75% of capacity retention; figs. S23 and S24). Whereas these performances certainly require additional electrode and electrolyte engineering to enhance the cycling stability and power performances, conceptually, it is interesting to compare the performances of Li_2_-BQDO to its closest analogue, the dilithium benzene-1,4-diolate (Li_2_-BQ). First, Li_2_-BQDO displays a higher redox potential (~300 mV) in both dissolved and solid forms (fig. S26) (*14*). This confirms the hypothesis proposed earlier that the induction of nitrogen between the hydroxyl redox center and the cyclohexadiene ring leads to enhanced electronic delocalization through resonance effects, as well as the redox potential increase. It is also worth noting that Li_2_-BQ can barely sustain few cycles in liquid electrolytes given the high solubility of the oxidized phase (1,4-benzoquinone) in polar solvents, whereas Li_2_-BQDO shows considerably ameliorated cycling stability, benefiting from the polymeric PNND phase impeding material dissolution. Using chemically synthesized PNND as active electrode material and starting the galvanostatic cycling from discharge (reduction) mode, similar galvanostatic profiles were attained (Fig. 3A, black curve, and fig. S25), additionally confirming that PNND polymer is the electrochemical oxidation product.

The large voltage hysteresis observed (reaching almost 1 V) in galvanostatic cycling profile of 1,2-oximate derivatives (**2**–**5**) triggered our interest in further analyses of this aspect, as this could be in line with previous mentions on the electrochemical irreversibility in 1,4-dioxime derivatives (*29*). Given that the simplest 1,2-oximate derivative (**3**) results in a liquid-phase product upon oxidation, extensive physicochemical characterization of the redox process reversibility was difficult to perform and therefore, lithiated diphenylglyoxime (Li_2_-DPGO, **2**) was chosen as prototype to explore the solid-state electrochemical behavior of lithiated 1,2-oximates. On galvanostatic charging (oxidation) of Li_2_-DPGO, the charge potential reaches a flat plateau around 3.1 V with efficient extraction of two electrons, reaching the theoretical capacity (212 mAh g^−1^; Fig. 3B, blue curve). The discharge and subsequent cycling were found to be reversible, proceeding through a two-electron/lithium transfer, yet the discharge was observed at a much lower potential (in average, at about 2.1 V versus Li^+^/Li), giving rise to a polarization of nearly 1 V. To confirm the reversibility of the chemical conversion process, the electrochemistry of the oxidized form of Li_2_-DPGO, namely, the DPODO, was also tested. The galvanostatic charge-discharge profile of DPODO active electrode material (given the oxidized state of DPODO, the cell was started in discharge-reduction mode) displayed comparable electrochemical cycling profile (Fig. 3B, black curve), confirming the chemical and electrochemical reversibility between Li_2_-DPGO and DPODO phases.

The reversible redox of the Li_2_-DPGO ⟷ DPODO and of other 1,2-oximate derivatives, conversion process consistently displayed high polarization (~0.6 to 1 V), persistent during subsequent charge-discharge cycles. Galvanostatic intermittent titration technique (GITT) analysis shows that the large part of polarization (~80%) originates from the oxidation (charge) process (fig. S28). As schematized in Fig. 2B, the open chain dioximate undergoes structural modification including oximate to nitroso oxidative conversion, *E*-*Z* configuration change, and furoxan cyclization. The electrode polarization is the result of many factors and parameters associated with the cell design (e.g., electrolyte conductivity, separator thickness, etc.), electrode design (carbon content, mass loading, etc.), and to potential intrinsic material redox kinetics. The insulator nature of Li_2_-BQDO and Li_2_-PADO indicates that electronic conductivity of electrode composite or the material itself does not contribute to this large voltage hysteresis (table S9). In organic compounds, polarization can also originate from molecular orbital rearrangements during redox, which may require additional energy, and thus result in high polarization or low Faradaic (energy) efficiency. In the 1,2-oximate case, assuming that the *E*-*Z* isomerization process as the limiting kinetic step in solid phase, and thus mainly responsible for the large polarization of the cells observed, we designed and studied the dilithium 9,10-phenanthrene dioximate (Li_2_-PADO, **4**) expected to bypass the *E*-*Z* isomerization given the molecularly locked configuration (fig. S29) and thus result in lower polarization.

However, high large hysteresis of ~1 V was also observed for the Li_2_-PADO active material, indicating that the *E*-*Z* isomerization is not the main contributor to the cell polarization (Fig. 3C). The experimental results have thus been corroborated to calculations for the energy profile of the Li_2_-DPGO ⟷ DPODO conversion process (analyzing both linear and closed structure reaction pathways and in both gas and solid phases). The analysis shows a dependence between structure type (linear or closed) and the oxidation state and that the structural change comes with an energy barrier of around 1 eV between reduced and oxidized states [Fig. 3E and refer to the Supplementary Materials for more density functional theory (DFT) details]. The orbital rearrangement requires expense of energy, which is reflected in the form of redox hysteresis or polarization. The computational results also indirectly explain the absence of polarization in 1,4-oximate (e.g., Li_2_-BQDO), which is due to minimal or no structural changes during the redox process. Moreover, small-energy barrier posed by availability of more than one coordination modes of Li-ions is escaped by the spontaneity of PNND polymer formation. The polymerization is also energetically favorable due to low enthalpy and high negative entropy of activation (−179.7 to −183.0 J K^−1^ mol^−1^) (*34*).

Unlike carbonyl-based redox systems, the conjugated oximates family is not limited to two-electron redox chemistry. By adopting well-defined molecular engineering methodologies, multiple oximate units have been further incorporated onto a single hydrocarbon backbone with higher capacity attained. The alicyclic α-oximate [Li_4_-TMTO (**5**)] with four oximate groups reaches a four-electron redox, with a theoretical specific capacity of 311 mAh/g. Li_4_-TMTO as active material displays a charge plateau at 3.5 V (versus Li^+^/Li) accompanied by a discharge process at an average potential of 2.65 V (versus Li^+^/Li; Fig. 3D). The average redox potential of Li_4_-TMTO (~3.0 V versus Li^+^/Li) is approximately 400 mV higher than that of Li_2_-DPGO (~2.6 V versus Li^+^/Li), Li_2_-DMGO (~2.6 V versus Li^+^/Li), and Li_2_-PADO (~2.6 V versus Li^+^/Li), which can be explained by the decrease of electron density due to the electron-withdrawing inductive effect (─I) of the four existing ─OCH_3_ groups.

### Structural and chemical reversibility

**Fig. 4.**
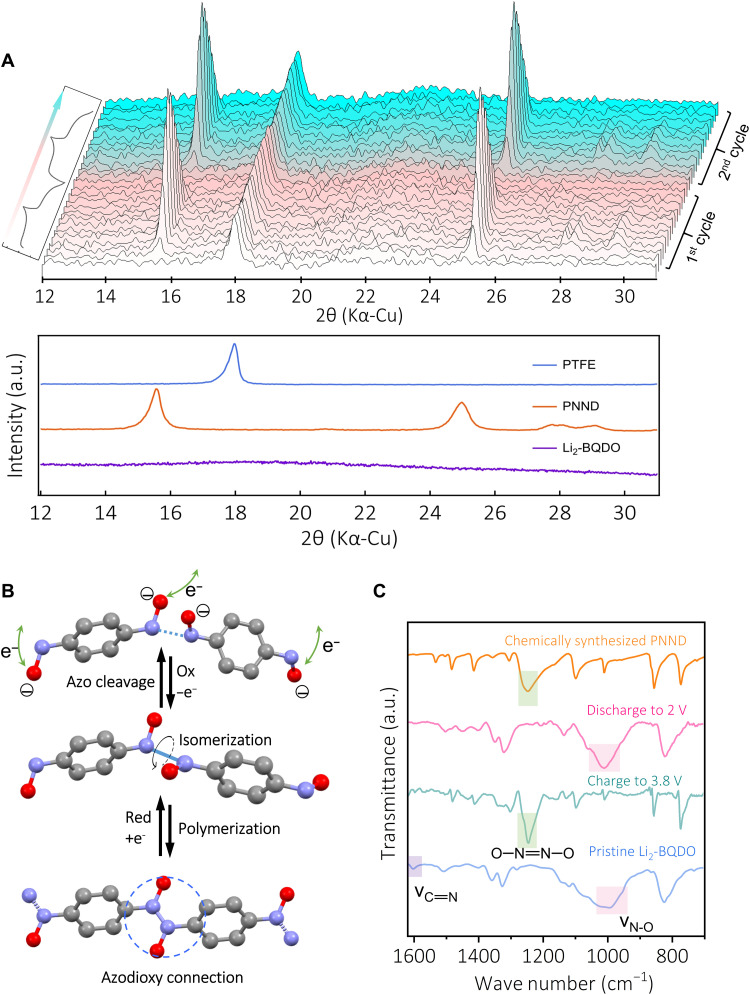
In situ and ex situ survey of the Li_2_-BQDO ⟷ PNND conversion process in solid-phase. (**A**) Top: In situ XRD of Li_2_-BQDO electrochemical conversion over the course of two full galvanostatic cycles. Bottom: Overlayed PXRD patterns of PTFE binder used for the in situ cell construct, as well as of pure, chemically synthesized Li_2_-BQDO and PNND phases. The *x* axis (2Θ, Kα-Cu) is common to both top and bottom. a.u., arbitrary unit. (**B**) Schematic illustration of the in situ electrochemical polymerization-depolymerization process. (**C**) Ex situ FTIR survey of the Li_2_-BQDO ⟷ PNND conversion process acquired at different charge states: pristine, fully charged to 3.8 V (corresponding to electrochemically generated PNND), and fully discharged to 2 V (corresponding to electrochemically generated Li_2_-BQDO). For comparison, the FTIR pattern of chemically synthesized PNND is also displayed.

The reversible electrochemical polymerization, depolymerization of the Li_2_-BQDO ⟷ PNND, or cyclization of the Li_2_-DPGO ⟷ DPODO processes, all accompanying the charge storage in solid phase, have been also monitored by operando XRD and ex situ infrared spectroscopy (Fig. 4 and figs. S30 to S32) to confirm the structural and chemical reversibility but also to attempt to further understand the dynamics of these processes in solid phase. While the solvated Li_2_-BQDO•8CH_3_OH intermediated was crystalline, the desolvation process (necessary for the electrochemical tests) resulted in the formation of an amorphous Li_2_-BQDO phase (Fig. 4A and fig. S31). Interesting, during the charge (oxidation) of Li_2_-BQDO, the progressive appearance of the crystalline PNND phase was observed, which was characterized by the main diffraction peaks at 15.5° and 25°, corresponding to (020) and (011) reflections plane respectively in the PNND phase (Fig. 4A and fig. S31). These observations clearly corroborate the fact that the product of solid-phase electrochemical oxidation of Li_2_-BQDO is PNND, with the transient formation of 1,4-dinitrosobenzene, that undergoes polymerization as discussed earlier. It should be mentioned that the molecular dinitroso form exists in dissolved phase in low amounts (see earlier discussion on results of chemical analysis and solubility of PNND). However, the coexistence of this form in solid phase at room temperature remains highly unlikely, where trace amounts remain undetectable by available techniques, according to previous studies and current findings.

During the subsequent reduction-depolymerization process (i.e., discharge/lithiation), the XRD peaks (of PNND phase) progressively vanished, with complete amorphization of the active material observed at the end of this step. This process was found reversible during the second full cycle (Fig. 4A). Although the phase structure of the Li_2_-BQDO could not be determined from these data nor that the same amorphous phase is generated upon electrochemical reduction (at the end of the two full galvanostatic cycles), we hypothesize that the Li_2_-BQDO ⟷ PNND interconversion is a synchronous biphasic process with the structural reversibility of the PNND phase. Additional support to this is also provided by the flat-plateau charge-discharge galvanostatic profiles (Fig. 3 and fig. S25), characteristic of biphasic reactions. It has to be however specified that this mechanistic view remains valid only when treating the equilibrium phases and is therefore insufficient as it does not take into account the transient dinitroso intermediate, with direct investigation of the dynamic process being required for this.

Given the ambiguity of the phase reversibility upon redox, the chemical reversibility of the redox-driven solid-phase polymerization was additionally confirmed by ex situ FTIR analysis (Fig. 4C). On oxidation, the characteristic ν_N─O_ and ν_C═N_ bands of Li_2_-BQDO disappeared, accompanied by the appearance of *E*-azodioxy signature (1264 cm^−1^), identical to the chemically synthesized PNND phase. The characteristic FTIR band for *Z*-azodioxy conformer (1350 to 1400-cm^−1^) was not detected; hence, this configuration can be excluded in the electrochemically generated PNND. Upon subsequent discharge, the FTIR spectrum was found to be closely similar to that of the pristine Li_2_-BQDO (Fig. 4C), further confirming the reductive depolymerization to Li_2_-BQDO. On similar lines, in situ XRD and ex situ FTIR survey of the electrochemistry in solid phase of α- or ortho-dioximates (e.g., Li_2_-DPGO ⟷ DPODO) also revealed the complete chemical and structural reversibility between the reduced (oximate form, Li_2_-DPGO) and oxidized compounds (furoxan form, DPODO) (fig. S30 and S32). These findings not only unravel the standing issue of reversibility of the electrochemical conversion between oximates and azodioxy or furoxan forms but also give guidelines for future researchers to unambiguously design new oximate cathodes and redox pathway to fully achieve the potential of the materials.

Although it is rarely observed with organic cathode materials, this type of reversible structural transformation (polymerization-depolymerization) during solid-state electrochemical processing remains exclusive and highly efficient in oximate-based redox systems. Electrochemical polymerization of redox systems followed by their utilization as battery active electrode materials is a relatively well-known process and has been largely applied with conducting polymers [polyaniline, poly(3,4-ethylenedioxythiophene) polystyrene sulfonate, and other] (*42*, *43*). There are also more exotic examples, such as the in situ electropolymerization of 4,4′,4″-tris(carbazol-9-yl)-triphenylamine give the ability of carbazole groups to polymerize under an electric field and subsequently used as a *p*-type high-voltage redox-active charge storage material (*44*). There are nevertheless subtle differences between the case of Li_2_-BQDO ⟷ PNND (disclosed here) and prior publications. The process presented in this work is a reversible biphasic electrochemical polymerization-depolymerization process, with synchronous *n*-type charge storage, whereas previous studies rely on distinct two-step processing, consisting of initial irreversible electropolymerization, followed by the use of thus formed polymeric material for *p*-type charge storage.

### Material-level energy metrics evaluation and future perspectives

**Fig. 5.**
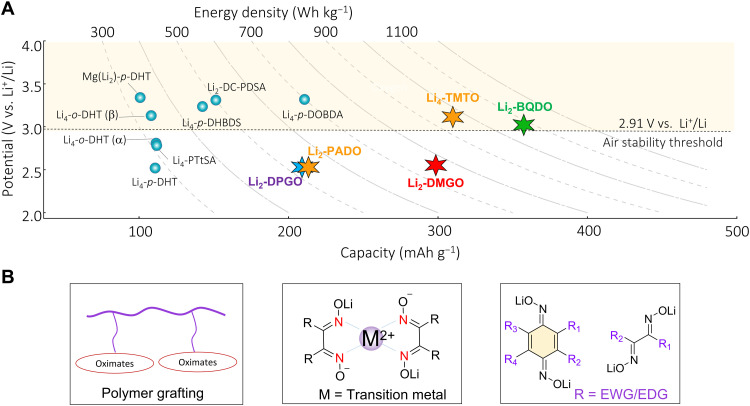
Material-level energy metrics and future perspectives of oximate materials for energy storage. (**A**) Reversible capacity, average redox potential, and the corresponding energy density of oximates (**1–5**, marked with a star symbol) as compared to state-of-art *n*-type organic positive electrode materials [Li_4_-o-DHT (α) (*6*), Li_4_-p-DHBDS (*11*), Mg(Li_2_)-p-DHT (*12*), Li_4_-DOBDA (*14*), Li_4_-o-DHT (β) (*15*), Li_4_-PTtSA and Li_2_-DC-PDSA (*16*), and Li_4_-*p*-DHT (*50*, *51*)]. The dashed line indicate the air stability threshold, 2.91 V versus Li^+^/Li, the redox potential at which oxygen reduction occurs versus Li^+^/Li redox couple. (**B**) The inspiration to improve the cell practical performance can be drawn from the rich family of carbonyl battery electrodes, including polymer side-chain grafting, coordination chemistry approaches (coordination polymers, metal-organic frameworks), or functionalization with electron withdrawing-donating groups for redox potential tuning and solubility limitations.

Although cycling stability remains still of concern for majority of organic battery materials, elevated redox potential (~3 V versus Li/Li^+^) combined with the high capacity (>300 mAh g^−1^) achieved with few oximate-based positive electrode materials are encouraging, considering the fundamental level of their technological development. The combination of high capacity and redox potential of this class of organic positive electrode materials paves the realization of long-pending goal of verging the energy density range of 1 kWh kg^−1^ (energy metric established at the material level), establishing superiority over the existing conjugated enolates, conjugated sulfonamides, and other organic battery material classes (in the range of 300 to 700 Wh kg^−1^; Fig. 5A and table S5). Although this value stands considerably higher than the energy content of many conventional inorganic positive electrode materials, it is also too early to consider the fundamental developments of this first generation of conjugated oximate battery electrode materials for commercialization due to the limited cycling performance (fig. S23). Further improvement of the overall performance can be achieved by various molecular architecture design, which could be inspired from the rich knowledge of quinone analogs (Fig. 5B) (*45*, *46*). For example, oximate redox centers can be incorporated within polymeric backbone wherein the distinct redox features can still be achieved with improved cycling stability. The α-dioximates are also known to act as chelating groups to transition metal ions forming coordination complexes or metal-organic frameworks (*47*). This could be also exploited and could potentially lead to redox potential increase through cation inductive effect and capacity increase if the redox of the metal ion can be accessed (*12*, *18*). The conjugated oximates also offer the possibility of redox potential tuning and solubility tuning by functionalizing with electron withdrawing/donating groups or anionic groups (e.g., methoxy, carboxylate, or sulfonate, as for instance discussed for Li_4_-TMTO) and the chemical space of conjugated oximate electrode materials is by no means limited to the five examples investigated in this work. Since oxime function can be easily accessed by the reaction of hydroxylamine with carbonyl derivatives, it is not excessive to assume that many of the quinone derivatives reported so far can be directly adapted to oximates, thereby leading to extending the library of organic Li-ion positive electrode materials.

The reversible redox of conjugated oximates in solid phase is revealed and shown to fulfill the requirements for performant Li-ion storage. When compared to carbonyl-based redox systems, unique molecular design and redox mechanism of the oximate groups offer net advantages in terms of dry air stability, increased energy content, and higher structural versatility, allowing the implementation of both cyclic and acyclic derivatives as electrode materials. All studied chemistries undergo synchronous one-electron redox per oximate unit to form nitroso derivatives, followed by major yet reversible structural modifications, including polymerization-depolymerization for cyclic 1,4-dioximates (e.g., Li_2_-BQDO, **1**) or intermolecular furoxan cyclization for alicyclic 1,2-dioximates (**2**–**4**). According to design rationales (Fig. 1), the oximate chemistries display high redox potential (2.5 to 3.2 V versus Li^+^/Li) and are stable toward oxidation in dry air, important aspects to consider for practical Li-ion positive electrode materials. The low molecular mass results in high specific capacities attained (in the range of 210 to 360 mAh g^−1^), which translates energy content at par with current technologies and materials. Creeping toward practical organic batteries, the versatility of organic chemistry for further molecular engineering and design of oximate family provides umpteen opportunities to further upgrade the cell parameters such as redox potential, capacity, and cycling stability.

## MATERIALS AND METHODS

1,4-Benzoquinone dioxime (95%, TCI Chemicals), dimethylglyoxime (98%, TCI Chemicals), anti-diphenylglyoxime (97%, Sigma-Aldrich), and DPODO (Sigma-Aldrich) were used as received. Lithium bis(trifluoromethylsulfonyl)imide (LiTFSI), ethylene carbonate (EC), dimethyl carbonate (DMC), and tetragylyme were obtained from Dodo Chem, China. Anhydrous solvents (THF, DMF, MeOH, and diethyl ether) for synthesis were purchased from Across Organics and used as received.

FTIR spectroscopy was carried out on Agilent Technologies Cary 630 FTIR operated in transmission or ATR mode. Nuclear magnetic resonance spectra were recorded with a Bruker magnet system 300 MHz/54 mm ultrashield spectrometer. Dimethyl sulfoxide-d6 was used as solvent and internal standard for chemical shifts. XRD patterns were collected on STOE DARMSTADT Transmission diffractometer system using Cu/Mo Kα1 radiation with a wavelength of 1.540600/0.70930 Å. Ultraviolet-visible spectroscopy studies were carried out using a Shimadzu UV-1700 PharmaSpec. Scanning electron microscopy (SEM) images were recorded on a JEOL FEG SEM 7600F (JEOL, Tokyo, Japan) Zeiss Neon 40 cross-beam workstation with Gemini SEM column (Carl Zeiss Iberia, S.L, Madrid, Spain). High resolution mass spectrometry (HR-MS) was measured using Thermo Scientific Q-Exactive Orbitrap mass spectrometer and APCI+ as ionization mode. The gel permeation chromatography (GPC) was performed on an Agilent GPC system equipped with an Agilent 1100/1200 pump (25°C; eluent, DMF; 2.5 mM NH_4_PF_6_; flow rate, 1 ml/min).

### Elemental analysis (CHNS) was done with Thermo Scientific FlashSmart elemental analyzer

#### X-ray crystallography

Single crystal x-ray data collection on Li_2_-BQDO-8MeOH was carried out on a Xcalibur, Ruby, Gemini ultra-instrument using Cu-Kα radiation (1.54184 Å) generated by a fine-focus sealed x-ray tube. Before measurement, the crystal was flash cooled to 100 K in an N_2_ gaseous stream. Data acquisition, reduction, and analytical face-index–based absorption correction were made using the program CrysAlis (*48*) and the implemented absorption correction was applied. The structure was solved by SHELXT and refined by SHELXL2014/7 (*49*). Anisotropic displacement parameters were applied for all atoms, except hydrogen atoms. All nonhydrogen atoms were refined anisotropically and H atoms were placed on calculated positions and refined in riding mode on their parent atoms with isotropic temperature factors, fixed at 1.2 Ueq of the parent atoms. CCDC number 2193545 (for Li_2_-BQDO-8MeOH) contains the supplementary crystallographic data for this paper. These data can be obtained free of charge from The Cambridge Crystallographic Data Centre via www.ccdc.cam.ac.uk/structures.

### Cyclic voltammetry

Cyclic voltammetry was conducted with a BioLogic Science Instruments SP-300. A three-electrode setup with screen-printed platinum as working and counter electrode and screen-printed Ag as the pseudo-reference electrode was used for measurements. A solution of 0.1 M LiCl in dimethyl sulfoxide was used as supporting electrolyte. Typically, 2 mM of the active material was dissolved in the electrolyte solution, and the cyclic voltammetry was measured at a scan rate of 100 mV s^−1^. After the respective scan, 2 mM ferrocene was added to the solution and used as an internal reference for redox potential determination.

### Cell assembly and testing

The electrodes were prepared by hand mixing and grinding the active material (50 wt %), conductive carbon (40 wt %), and polytetrafluoroethylene (PTFE) binder (10 wt %) for 15 min. The formed powder mixture was added onto the positive side of a coin cell case and pressed with a stainless-steel disk. A typical mass loading of 4 mg/cm^2^ was used. The electrochemical properties and galvanostatic charge/discharge tests were performed in 2032-type coin cells with 13-mm lithium chips as the counter and pseudo-reference electrodes, one sheet of glass microfiber filter (Whatman GF/D, Aldrich) as separator, with different electrolyte formulations: 5 M LiTFSI in tetraglyme and 7 M LiTFSI in EC/DMC. Galvanostatic charge/discharge tests were performed on Neware battery testing system at ambient temperature. GITT was tested under a galvanostatic current pulse of C/10 for a duration of 2 hours followed by relaxation at open circuit for 1 hour at each step.

### In situ XRD battery measurement

In situ XRD measurement was performed on STOE DARMSTADT Transmission diffractometer system using Cu Kα1 radiation with a wavelength of 1.540600 Å. The electrodes were prepared by hand mixing and grinding the active material (50 wt %), conductive carbon (40 wt %), and PTFE (10 wt %), pressed onto an Al mesh as current collector.

## Supplementary Materials

### This PDF file includes:

Supplementary Text Figs. S1 to S41 Tables S1 to S10 References
