# Supplementary material for: Revealing the reversible solid-state electrochemistry of lithium-containing conjugated oximates for organic batteries
Source: Sci Adv. 2023 Apr 28;9(17):eadg6079. doi: 10.1126/sciadv.adg6079 (PMC10146882; doi:10.1126/sciadv.adg6079)
Supplement: Supplementary file 1 — Supplementary Text Figs. S1 to S41 Tables S1 to S10 References [file sciadv.adg6079_sm.pdf]

Science Advances
AAAS

Supplementary Materials for

Revealing the reversible solid-state electrochemistry of lithium-containing conjugated oximates for organic batteries

Jiande Wang *et al.*

Corresponding author: Deepak Gupta, deepakg2003@gmail.com; Alexandru Vlad, alexandru.vlad@uclouvain.be

Sci. Adv. **9**, eadg6079 (2023)
DOI: 10.1126/sciadv.adg6079

This PDF file includes:

Supplementary Text
Figs. S1 to S41
Tables S1 to S10
References

Materials Synthesis and Characterization

• Dilithium dimethylgyloxime (Li_2-DMGO)

Dimethylgyloxime (1.16 g, 10 mmol) was dissolved in anhydrous methanol (20 ml) and Lithium methoxide (760 mg, 20 mmol) was added to the solution under stirring. The reaction (performed in an Argon filled glove box), was allowed to stir for 24 h at room temperature. The yellow reaction mixture was poured into 100 mL of anhydrous diethyl ether to precipitate the product, followed by filtration, washing with copious amounts of diethyl ether and drying at 60 °C under vacuum. The product (Li_2-DMGO) was obtained in quantitative yield.

- ^{1}H NMR (300 MHz, MeOD) δ 2.01 ppm. ^{13}C NMR (75 MHz, MeOD) δ 154.95, 8.38 ppm.

- HRMS (ESI) calcd for $C_4H_6Li_2N_2O_2$: 128.0749, Found: 127.9790 $[M]^+$.

- Elemental Analysis: Calculated: C: 37.54%; H: 4.73%; N: 21.89%. Found: C: 37.38%; H: 5.03%; N: 20.10%.

Supplementary Figure S1. ^{1}H NMR (MeOD) spectrum of Li_2-DMGO.

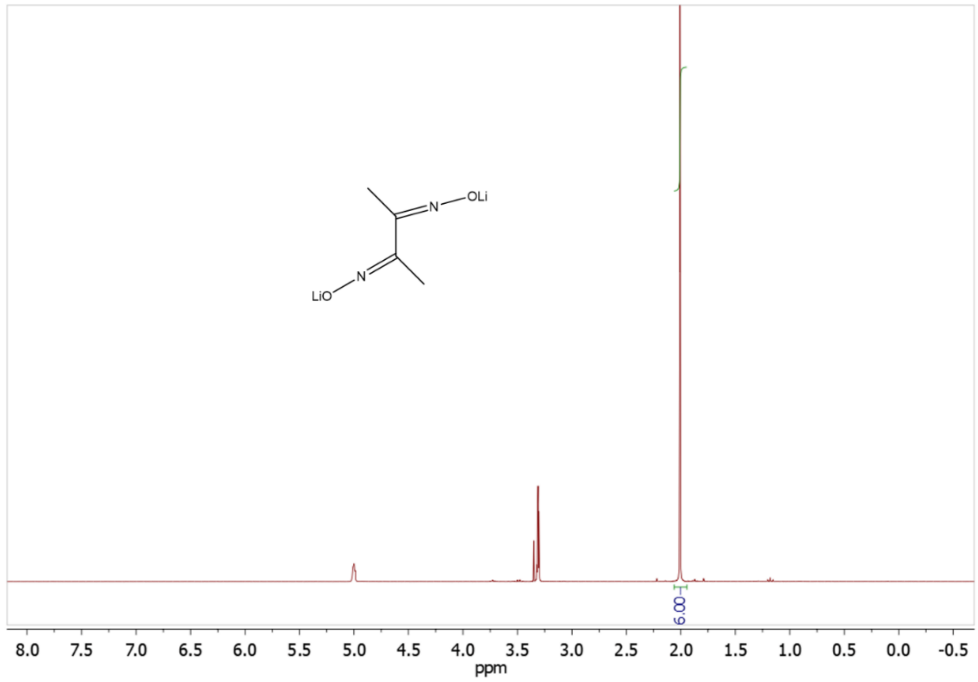

Supplementary Figure S2. ^{13}C **NMR (MeOD) spectrum of** Li_2**-DMGO.**

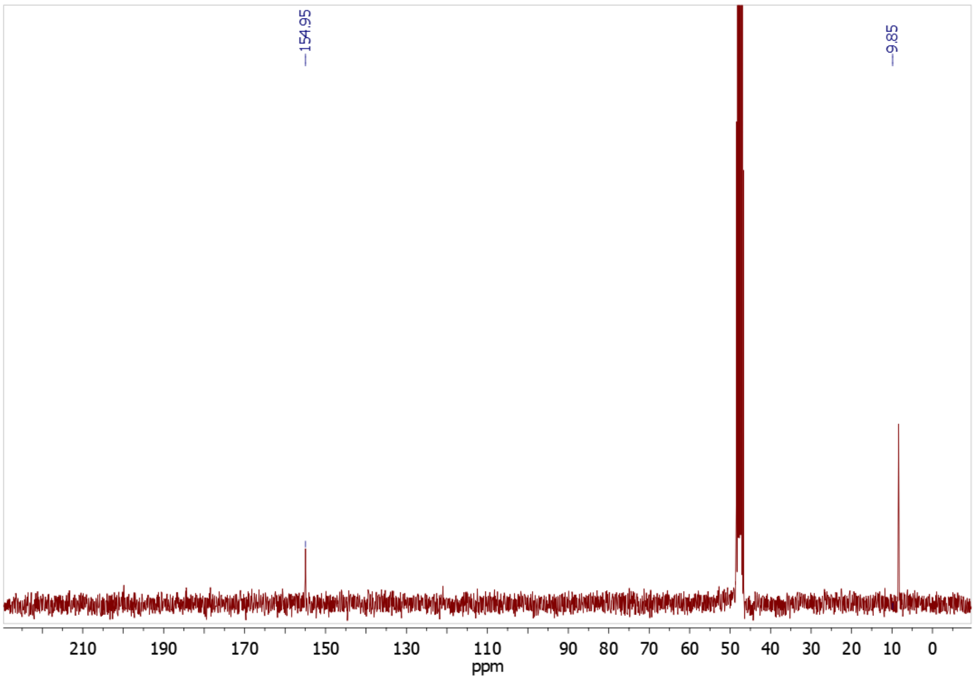

• **Dilithium p-Benzoquinone dioxime (Li_2-BQDO)**

p-Benzoquinone dioxime (276 mg, 2 mmol) was dissolved in anhydrous THF (6 ml), and solid lithium hydride (32 mg, 4 mmol) was added to the solution under stirring. The reaction (performed in an Argon filled glove box) was allowed to stir for 48 h at room temperature. The reaction mixture was afterwards poured into 6 mL of anhydrous diethyl ether to precipitate the product, followed by centrifugation and washed with a copious amount of diethyl ether. The product (Li_2-BQDO) was collected and dried under vacuum at 70 °C for 2 h and 200 °C for 12 h.

- ^{1}H NMR (300 MHz, MeOD) δ 7.25 – 7.01 (m, 2H), 6.77 – 6.55 (m, 2H).

- ^{13}C NMR (75 MHz, MeOD) δ 156.17, 155.87, 130.22, 126.21, 118.41, 115.68 ppm.

- HRMS (ESI) calcd for $C_6H_4Li_2N_2O_2$: 150.0593, found 151.0350 $[M-H]^+$.

- Elemental Analysis: Calculated: C: 48.05%; H: 2.69%; N: 18.68%. Found: C: 47.65%; H: 3.45%; N: 17.58%.

Supplementary Figure S3. ^{1}H NMR (MeOD) spectrum of Li_2-BQDO.

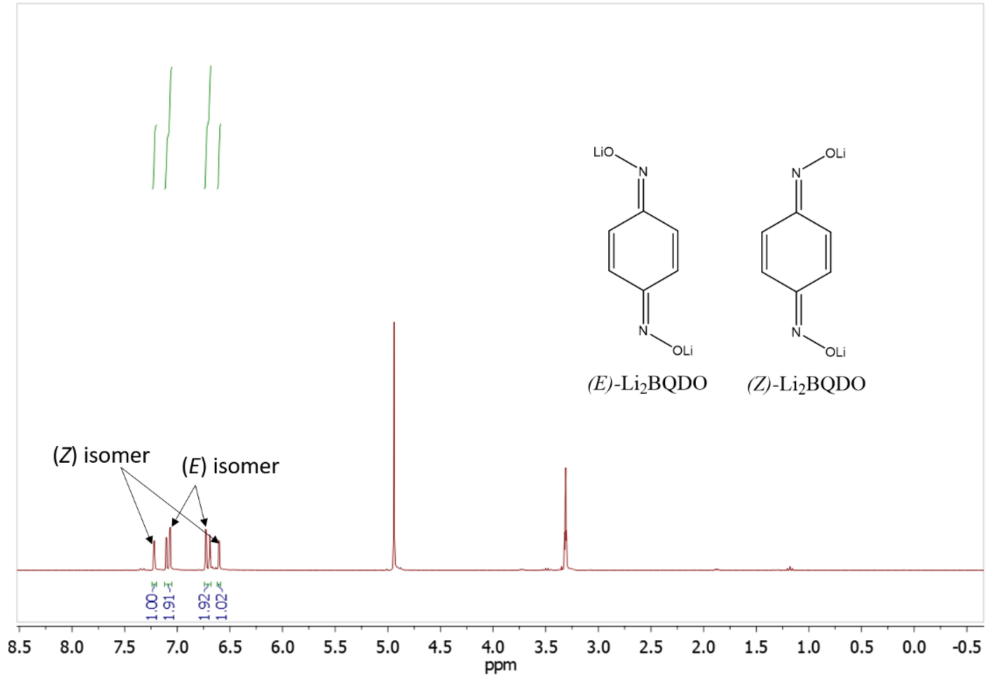

Supplementary Figure S4. ^{13}**C NMR (MeOD) spectrum of** Li_2**-BQDO.**

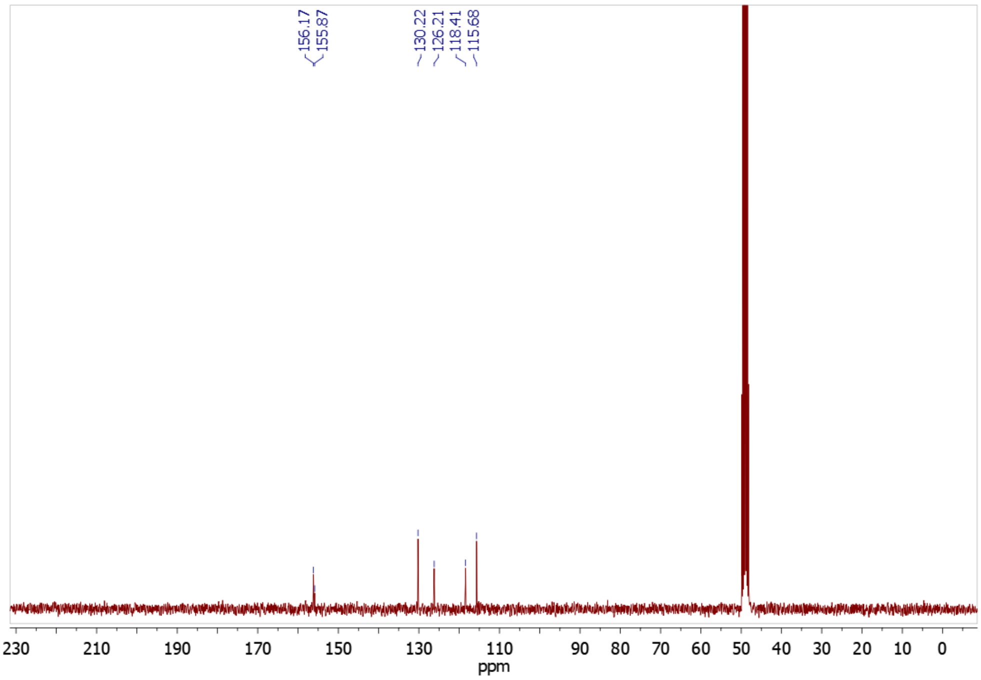

• Dilithium diphenylglyoxime (Li_2-DPGO)

Diphenylglyoxime (240 mg, 1 mmol) was suspended in a mixture of anhydrous tert-butyl alcohol (5 ml) and MeOH (5 ml), and lithium tert-butoxide (160 mg, 2 mmol) was added to the suspension under stirring. The reaction (performed in an Argon filled glove box) was kept for 24 h at room temperature. The light brown precipitate was then filtered and washed with diethyl ether (10 mL) 3 times. The product (Li_2-DPGO) was collected and dried at 60 °C, then at 200 °C for 2h under vacuum giving a yield of 90%.

- ^{1}H NMR (300 MHz, MeOD) δ 7.43-7.37 (m, 4H), 7.23 – 7.16 (m, 6H) ppm.

- ^{13}C NMR (75 MHz, MeOD) δ 156.86, 136.72, 129.24, 129.11, 128.80 ppm.

- HRMS (ESI) calcd for $C_{14}H_{10}Li_2N_2O_2$ 252.1062, found 252.9240$[M]^+$.

- Elemental Analysis: Calculated: C: 66.69%; H: 4.00%; N: 11.11%. Found: C: 65.67%; H: 3.77 %; N: 10.79 %.

Supplementary Figure S5. ^{1}H NMR (MeOD) spectrum of Li_2-DPGO.

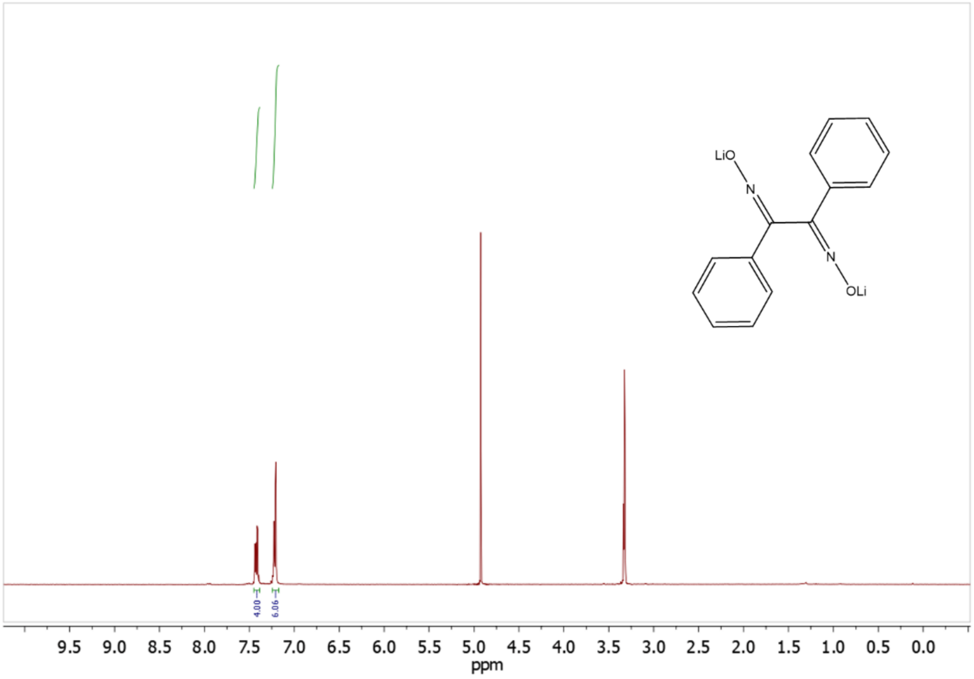

Supplementary Figure S6. ^{13}C NMR (MeOD) spectrum of Li_2-DPGO.

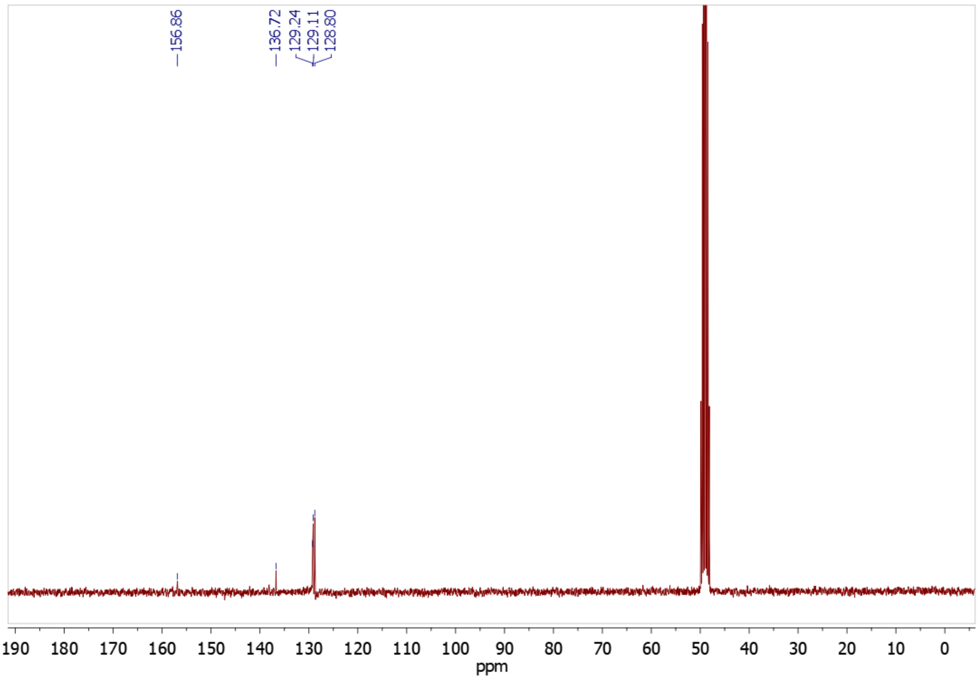

•Dilithium 9,10-phenanthrenedione oximate (Li_2-PADO)

To a suspension of 9,10-phenanthrenedione (2.08 g, 10 mmol) in EtOH (50 ml), a EtOH (30 ml) solution of $NH_2OH{\cdot}HCl$ (1.63 g, 25 mmol) and pyridine (2.03 ml, 25 mmol) was added. The resulting orange suspension was refluxed for 22 h, during which the solids dissolved, and the suspension turned to clear yellow solution. The resulting solution was cooled, filtered, and left for slow evaporation at room temperature. After 24 h, a yellow microcrystalline solid of the dioxime was formed, which was collected by filtration, washed with cold EtOH, and dried under vacuum for 24 h. The yield was 90%.

- ^{1}H NMR (300 MHz, DMSO-d6) δ 12.34 – 12.12 (m, 2H), 8.42-8.47 (m, 1H), 8.04-8.12 (m, 2H), 7.74-7.83 (dd, J = 7.7, 1.4 Hz, 1H), 7.38 – 7.55 (m, 4H) ppm.

- ^{13}C NMR (75 MHz, DMSO) δ 133.22, 132.26, 131.58, 130.76, 130.01, 128.81, 128.17, 127.94, 126.72, 125.04, 124.94, 124.74, 124.36 ppm.

Supplementary Figure S7. ^{1}H NMR (DMSO-d6) spectrum of H_2-PADO.

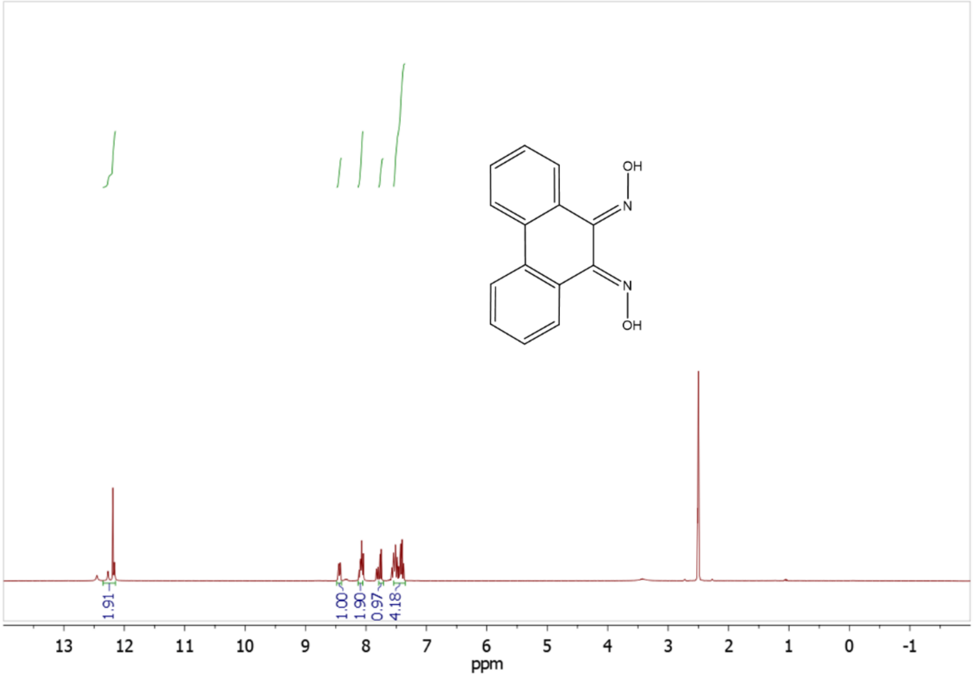

Supplementary Figure S8. ^{13}C NMR (DMSO-d6) spectrum of H_2-PADO.

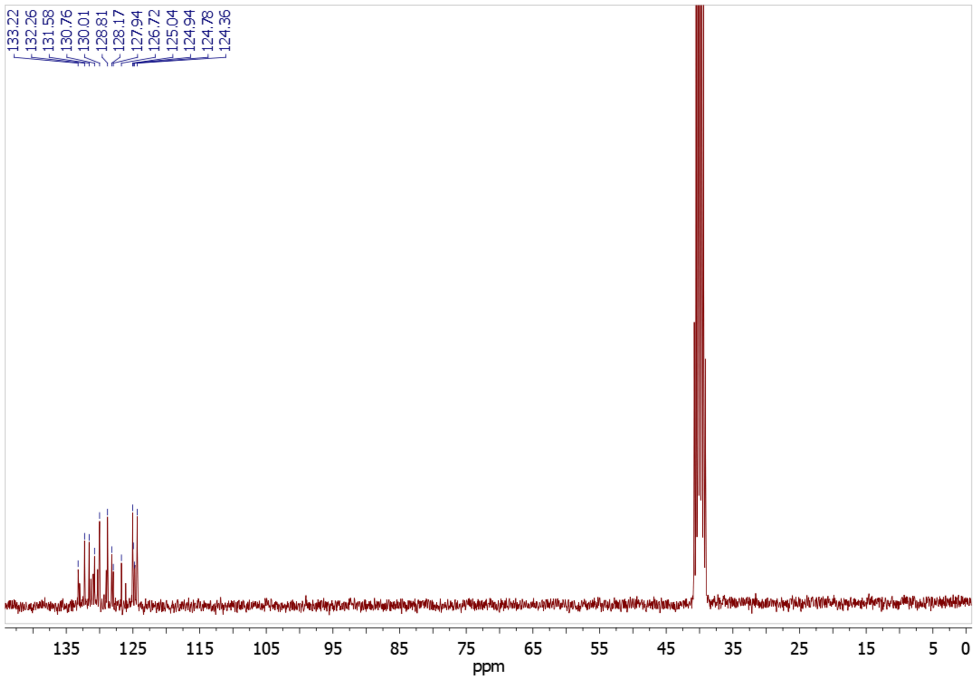

The lithiation was performed by suspending H_2-PADO (238 mg, 1mmol) in MeOH (5 ml). To the stirred suspension, lithium methoxide (76 mg, 2 mmol) was added. The reaction (performed in an Argon filled glove box) was kept for 24 h at room temperature. The resulting product was obtained by adding an excess amount of anhydrous diethyl ether (30 ml) to the solution. The product (Li_2-PADO) was collected by filtration, and dried at 60 °C under vacuum, followed by 200 °C under vacuum for 3 h.

- ^{1}H NMR (300 MHz, MeOD) δ 8.10 (dd, *J* = 7.9, 1.4 Hz, 2H), 8.03 (d, *J* = 7.6 Hz, 2H), 7.49 – 7.42 (m, 2H), 7.37 – 7.30 (m, 2H).

- ^{13}C NMR (75 MHz, MeOD) δ 152.59, 132.87, 132.29, 130.03, 128.83, 126.27, 124.29 ppm.

- HRMS (ESI) calcd for $C_{14}H_8Li_2N_2O_2$: 250.0906 $[M-Li]^+$, found 250.9920 $[M-H]^+$.

- Elemental Analysis: Calculated: C:67.23%; H: 3.22%; N: 11.20%. Found: C: 66.27%; H: 3.22%; N: 10.57 %.

Supplementary Figure S9. ^{1}**H NMR (MeOD) spectrum of Li$_2$-PADO.**

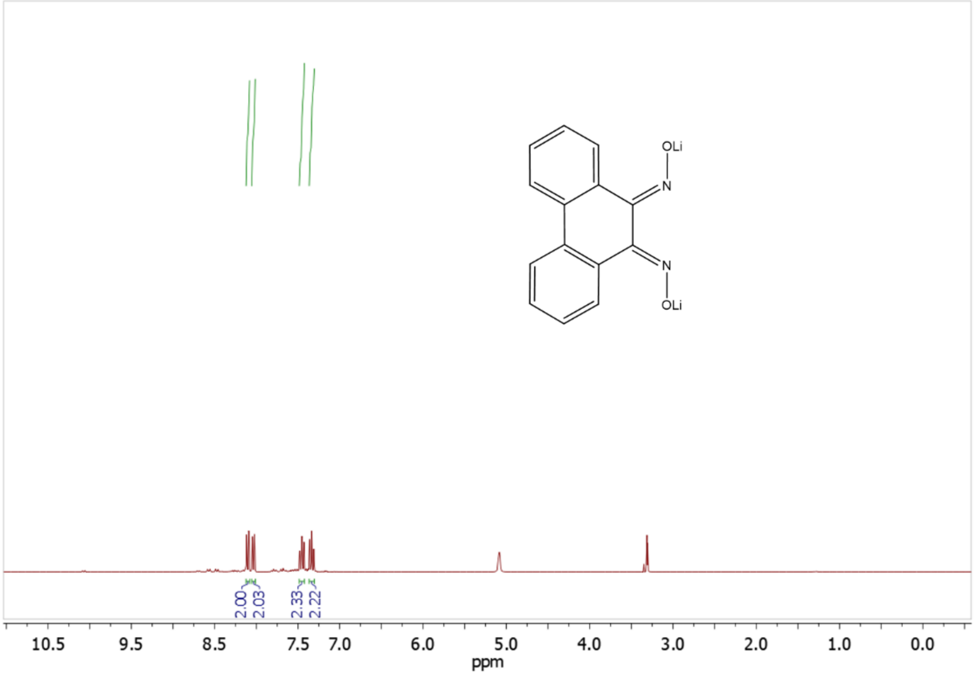

Supplementary Figure S10. ^{13}**C NMR (MeOD) spectrum of Li$_2$-PADO.**

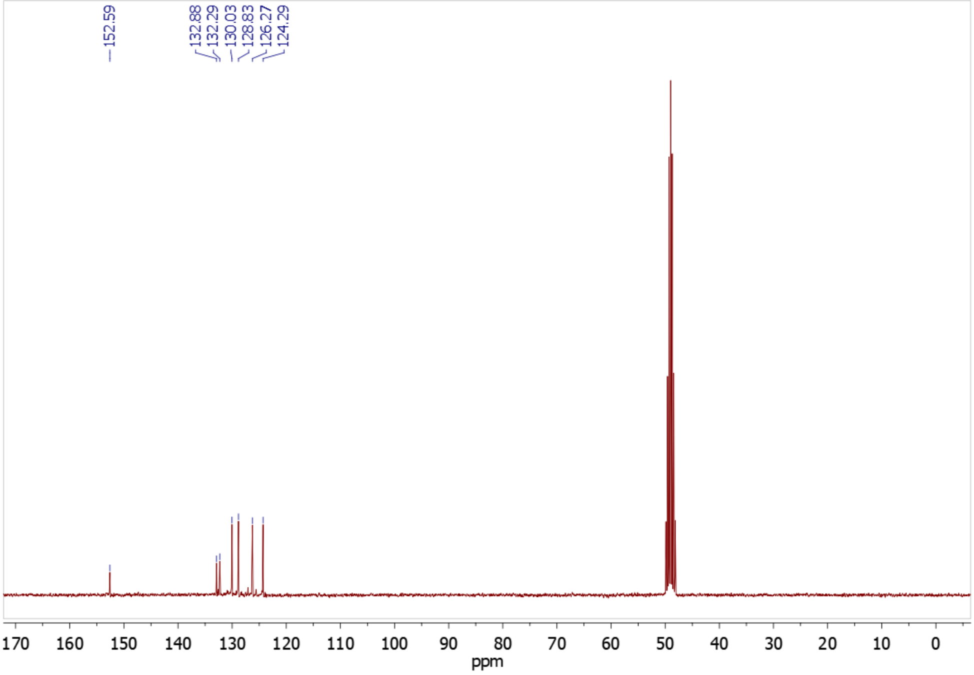

• **Tetralithium-1,1,4,4-Tetramethoxy-2,3,5,6-tetraoximatecyclohexane (Li_4-TMTO)**

To a solution of phloroglucinol dihydrate (10 g, 0.06 mol) in methanol (100 mL), 0.5 ml of acetic acid was added. The suspension was cooled to 0 °C, and isopropyl nitrite (22 mL, 0.214 mol) was added dropwise within 1h, while stirring and keeping the temperature of reaction at 0°C. The mixture was stirred for another 0.5 h at room temperature. After that, the solvent was evaporated in vacuo at 35 °C of the water bath. The crude product was suspended in diethyl ether, a solid was filtered off, washed with diethyl ether, and dried. The material was used for the next step without further purification (*52*).

The 2,4,6-Trinitrosobenzene-1,3,5-triol (12.8g, 0.055 mol) obtained above was solubilized in 200ml of methanol to which 3.84g of hydroxylamine hydrochloride was added. The red solution was kept at room temperature for 30 days, afterwards, the white precipitate was filtrated, washed with water, methanol and dried at room temperature. Yield: 11.5 g of H_4-TMTO, 65%.

- ^{1}H NMR (300 MHz, DMSO-d6): δ = 3.15 (6H, s, OMe), 3.41 (6H, s, OMe), 11.53 (4H, s, NOH) ppm.

- ^{13}C NMR (100 MHz, DMSO-d6): δ = 50.77 (OMe), 51.63 (OMe), 100.12 (C(OMe)), 144.95(C=NOH) ppm.

Supplementary Figure S11. ^{1}H NMR and ^{13}C NMR (DMSO-d6) spectra of H_4-TMTO.

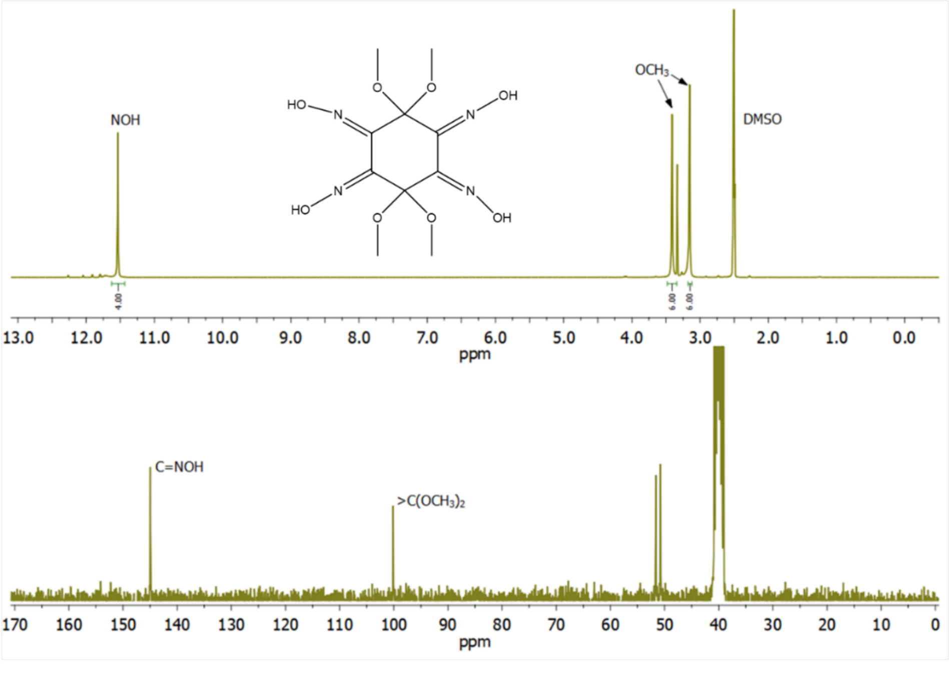

100 mg (0.31mmol) of H_4-TMTO was suspended in methanol (9ml) and lithium methoxide (48.5 mg, 1.27mmol) was added to the solution under stirring. The reaction (performed in an Argon filled glove box) was kept for 24 h at room temperature. Then, anhydrous diethyl ether (30 ml) was added to the reaction mixture, resulting in the precipitation of a white product, followed by filtration and washed with diethyl ether 4 times. The product (Li_4-TMTO) was dried at 120 °C for 12h under a vacuum to remove free diethyl ether and methanol. Yield: 80.5mg, 75%.

- ^{1}H NMR (300 MHz, MeOD): δ = 3.32 (6H, s, OMe) ppm.

- ^{13}C NMR (75 MHz, MeOD): δ = 51.60 (OMe), 101.31 (C(OMe)), 152.34(C=NOH) ppm.

- HRMS (ESI) calcd for $C_{10}H_{12}Li_4N_4O_8$: 344.1295, found: 345.0100$[M-H]^+$.

- Elemental Analysis: Calculated: C:34.92%; H: 3.52%; N: 16.29%. Found: C: 33.40%; H: 3.73%; N: 15.92 %.

Supplementary Figure S12. ^{1}H NMR (MeOD) spectrum of Li_4-TMTO.

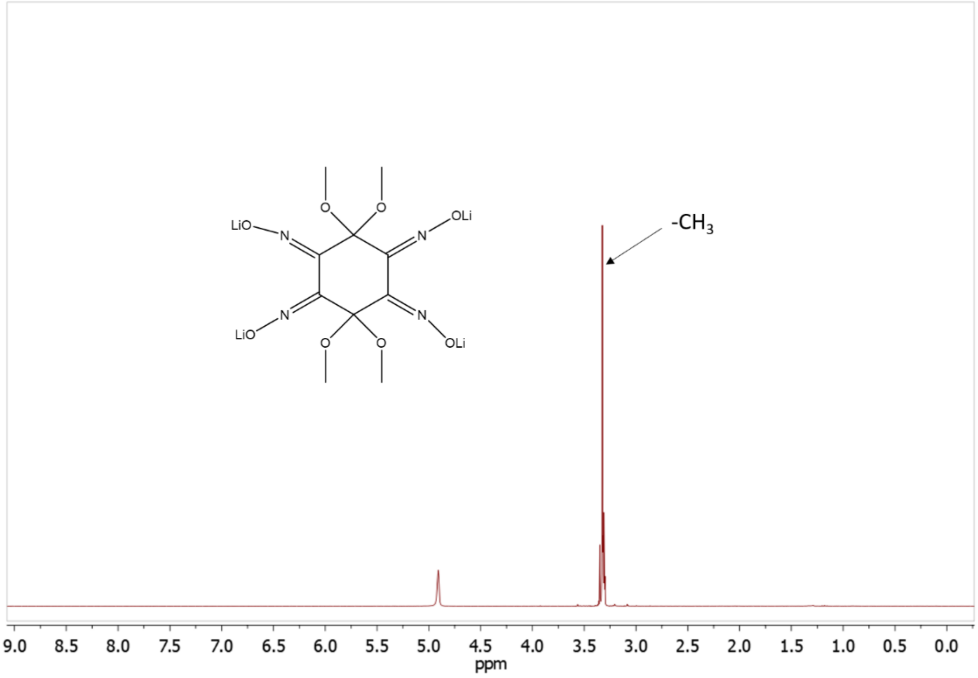

Supplementary Figure S13. ^{13}C NMR (MeOD) spectrum of Li_4-TMTO.

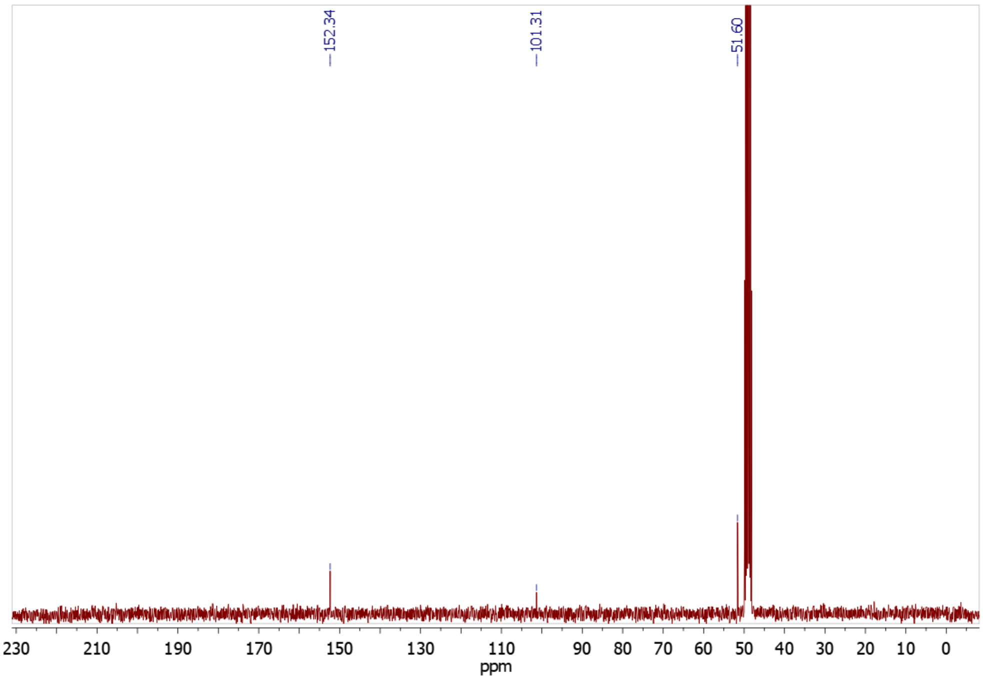

• Poly(1,4-phenyleneazine-N,N-dioxide) (PNND) (*53*)

To a stirred solution of 1.38 g p-benzoquinone dioxime (10 mmol) and 0.84 g sodium hydroxide (21 mmol) in 30 ml water, 30 ml of 5.5% sodium hypochlorite (excess amount) was slowly added. The solution turned yellow, and a yellow precipitate gradually formed. Stirring was continued for 1h. The precipitate was filtered and washed with water 3 times and acetone, followed by drying under vacuum at RT to form PNND, with a yield of 65%.

- Elemental Analysis: Calculated: C: 52.95%; H: 2.93%; N: 20.58%. Found: C: 52.70%; H: 2.91%; N: 19.46 %.

Crystal structure determination by X-ray powder diffraction method.

The crystal structure of PNND was solved from the powder XRD data. Powder X-ray diffraction (PXRD) patterns were collected on a STOE Stadi P diffractometer in transmission geometry equipped with a Cu anticathode (Kα radiation, λ = 1.540600 Å, operating at 50 kV – 40 mA). For the structure determination from PXRD, we followed the following steps: pattern indexing, space group determination, structure solution and the Rietveld refinement. The indexing procedure was carried out in EXPO2014 (*54*), and FullProf suite (*55*) using NTREOR, DICVOL. By these indexing procedures a monoclinic system was obtained with high figure of merit (F.O.M.). Space group determination, structure solution and Rietveld refinement were performed in EXPO2014.

Supplementary Table S1. Powder XRD data and refinement parameters of PNND[a]

Powder data	
Formula	C_3H_2NO
Powder Data temperature (K)	295
M_r	68.055
crystal system	Monoclinic
space group	$P2_1/n$
a (Å)	6.394(2)
b (Å)	11.322(4)
c (Å)	3.7072(12)
b (deg.)	92.923(8)
V (Å^3)	268.03(15)
Z	4
ρ (g cm^{-3})	1.687
Data collection	
λ (Å)	1.540600
q_{min}–q_{max} (deg.)	5-70
2*q* step (deg.)	0.015
Refinement	
Profile function	Pseudo-Voigt
R_p	2.27
R_{wp}	4.76
R_e	0.992
Chi^2 (X^2)	22.97
R (Bragg)	18.11
R (structure factor)	13.49

[a] *Structure is similar to the reported CCDC reference No. 2006601*

Supplementary Table S2. Fractional Atomic Coordinates (×10^4) and Equivalent Isotropic Displacement Parameters (Å^2×10^3) for PNND. U_{eq} is defined as 1/3 of the trace of the orthogonalised U_{IJ} tensor.

Atom	*x*	*y*	*z*	U(eq)
C9	5947	8903	10997	0(6)
C10	7126	9820	9598	0(7)
C11	6179	10897	8582	0(6)
N4	9303	9641	9188	0(4)
O2	9886	8732	7066	150(6)

Supplementary Table S3. Bond Lengths for PNND.

Atom	Atom	Length/Å	Atom	Atom	Length/Å
C9	C10	1.398377	C11	C9[1]	1.394392
C9	C11[1]	1.394390	N4	N4[2]	1.328292
C10	C11	1.405053	N4	O2	1.358861
C10	N4	1.421947			

Supplementary Table S4. Bond Angles for PNND.

Atom	Atom	Atom	Angle/°	Atom	Atom	Atom	Angle/°
C10	C9	C11[1]	117.93	C10	C11	C9[1]	121.36
C9	C10	C11	120.70	C10	N4	N4[2]	120.15
C9	C10	N4	118.89	C10	N4	O2	117.95
C11	C10	N4	120.40	N4[2]	N4	O2	121.89

Reversible redox-active moiety	Classification	General redox mechanism	Example of electrode reaction
Conjugated carbonyl	n-type	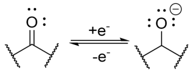	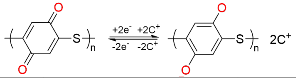
Organodisulfide	n-type	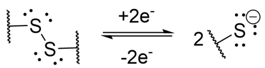	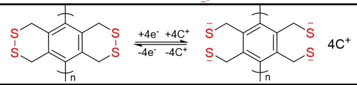
Conjugated azo group	n-type	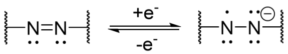	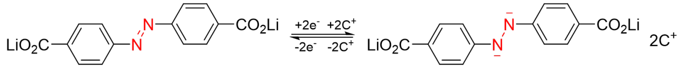
Conjugated nitrile	n-type	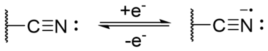	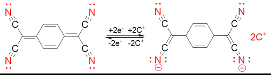
Conjugated amine	p-type	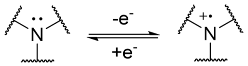	Ppy 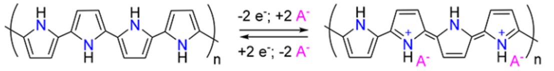
Conjugated etheroxide	p-type	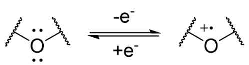	DBMMB 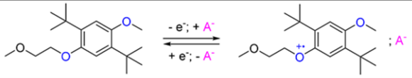
Conjugated thioether	p-type	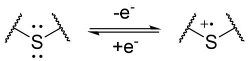	PT 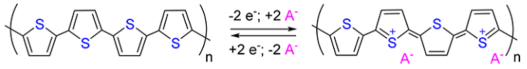
Nitroxide radical	n/p-type (bipolar)	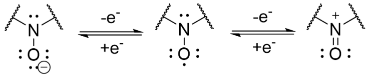	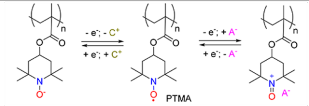
Conjugated sulfonamide	n-type	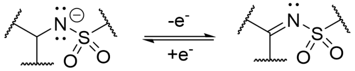	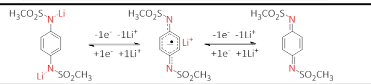
**Conjugated oximate (this work)**	**n-type**	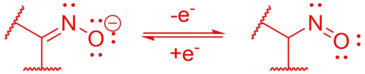	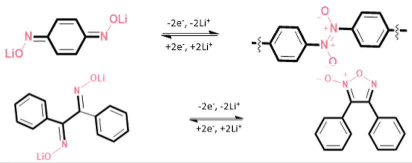

Supplementary Table S5. Reaction mechanisms and groups for electrochemical storage in reported redox-active organic materials, compared to the oximate chemistry disclosed in this work (highlighted in red). Table adapted with permission from ref (*1*). Copyright 2020 American Chemical Society. Colors are used to highlight the redox units.

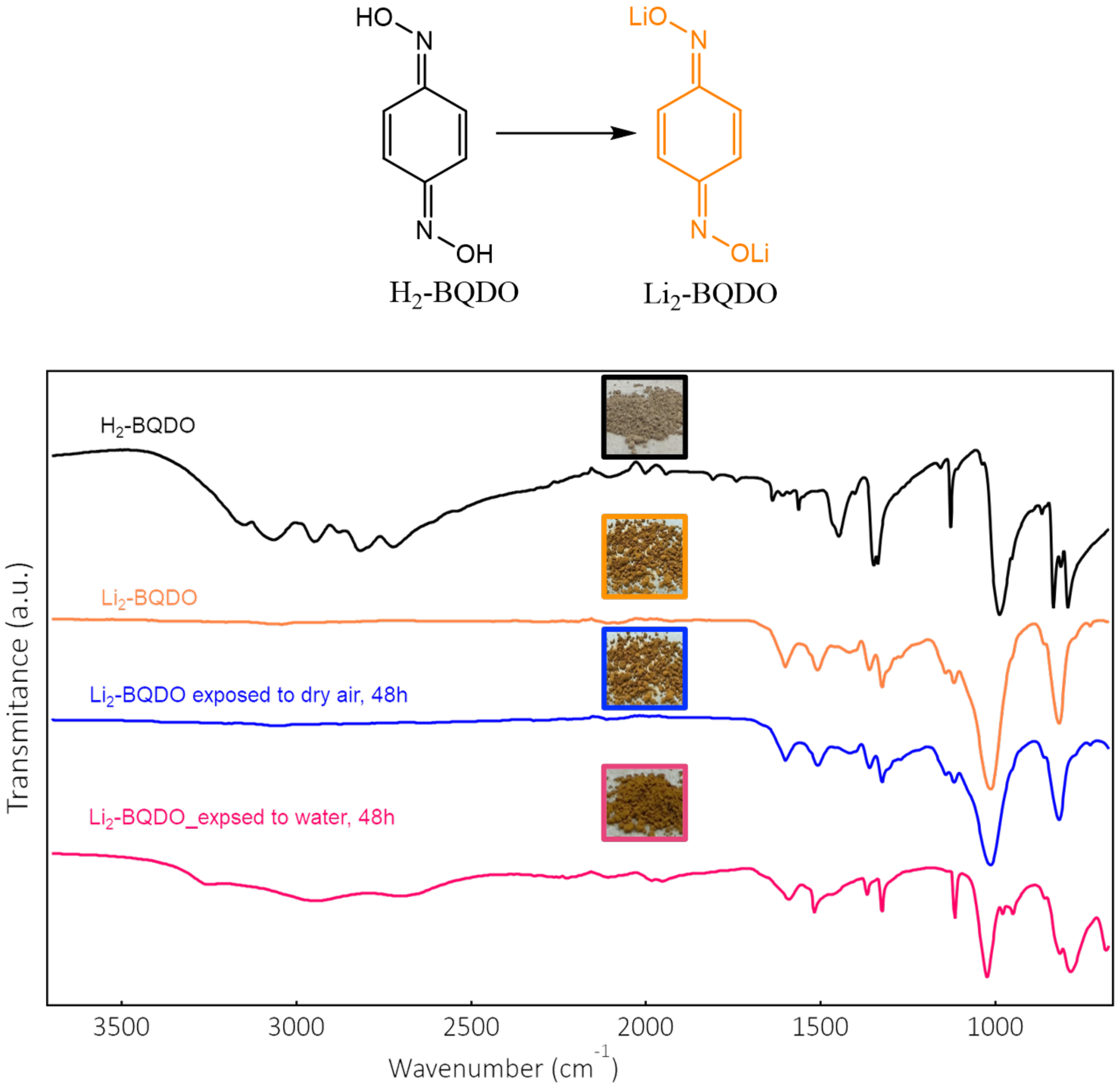

Supplementary Figure S14. FTIR analysis survey for the synthesis of Li_2-BQDO, coupled to air and moisture stability analysis.

The formation of lithiated product (Li_2-BQDO, orange curve) is confirmed by the disappearance of the weak broad band around 3000 cm^{-1} attributed to the hydroxyl groups of H_2-BQDO (black curve). After lithiation, the broad -OH band completely disappears (orange curve). Comparative FTIR spectra of pristine and dry air-exposed (for 48h) samples of Li_2-BQDO (blue curve) and water vapours exposed (for 48h) Li_2-BQDO (pink curve). The materials show identical FTIR signatures after dry air-exposure without any sign of decomposition, indicating excellent oxygen tolerance. For water vapor exposed Li_2-BQDO (pink curve), residual water or partial hydrolysis can be observed with the characteristic peaks still preserved after 48h of exposure.

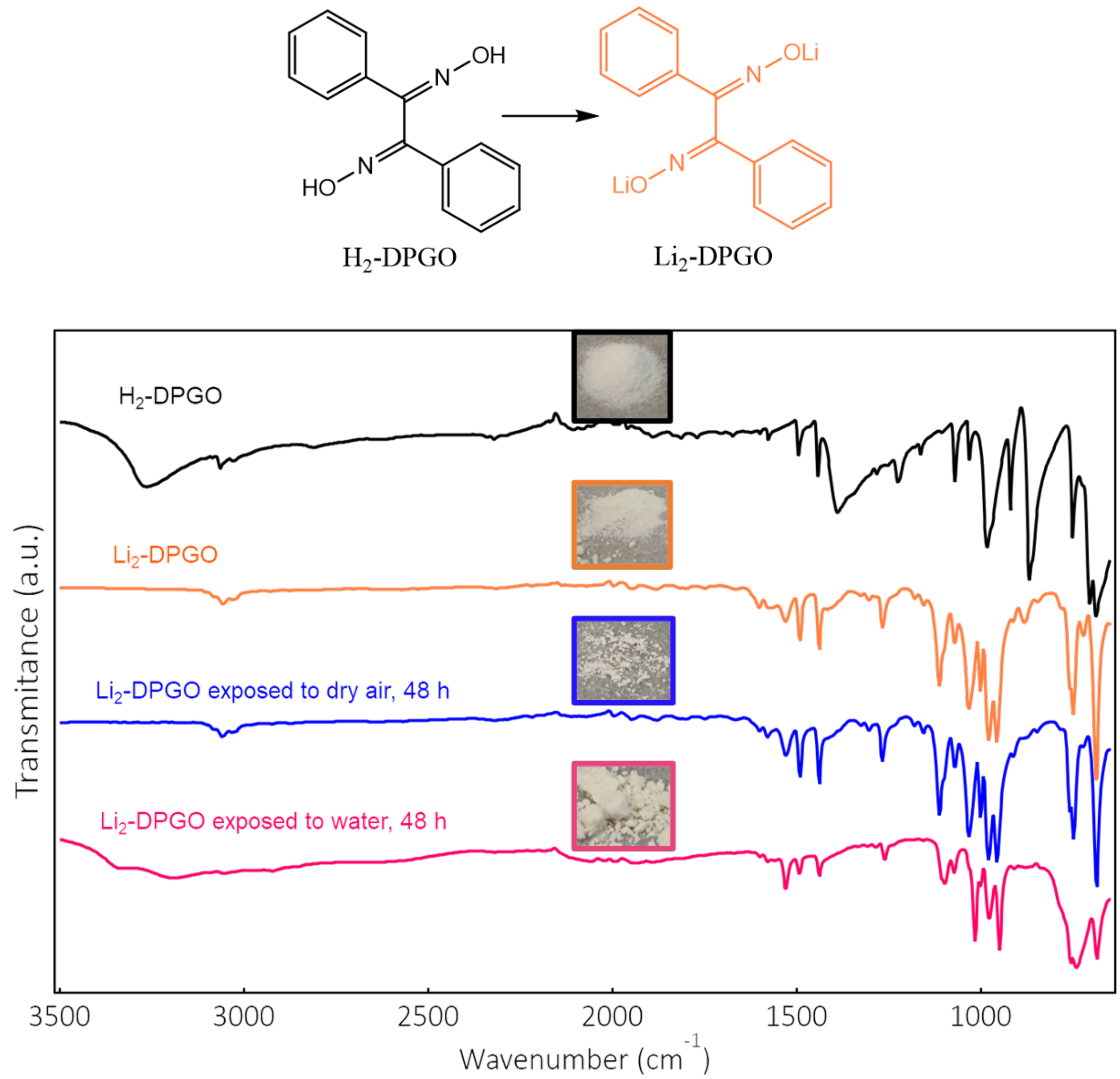

Supplementary Figure S15. FTIR analysis survey for the synthesis of Li_2-DPGO, coupled to air and moisture stability analysis.

The formation of pure Li_2-DPGO (orange curve) after lithiation of H_2-DPGO (black curve) is confirmed by the disappearance of weak and broad O-H stretch band between 3200-3300 cm^{-1}. Comparative FTIR spectra of pristine and dry air-exposed (for 48h) samples of Li_2-DPGO (blue curve) and water vapours exposed Li_2-DPGO (pink curve, for 48h). The materials show identical FTIR signatures after dry air-exposure without any sign of decomposition. For water vapour exposed Li_2-DPGO (pink curve), residual water or partial hydrolysis can be observed with the characteristic peaks still preserved after 48h of exposure.

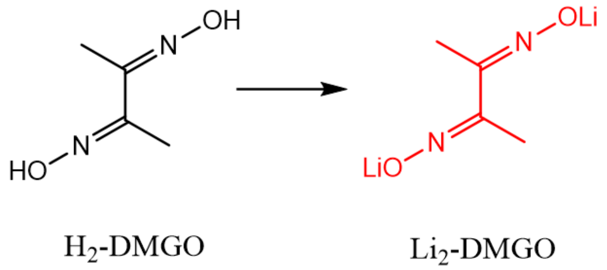

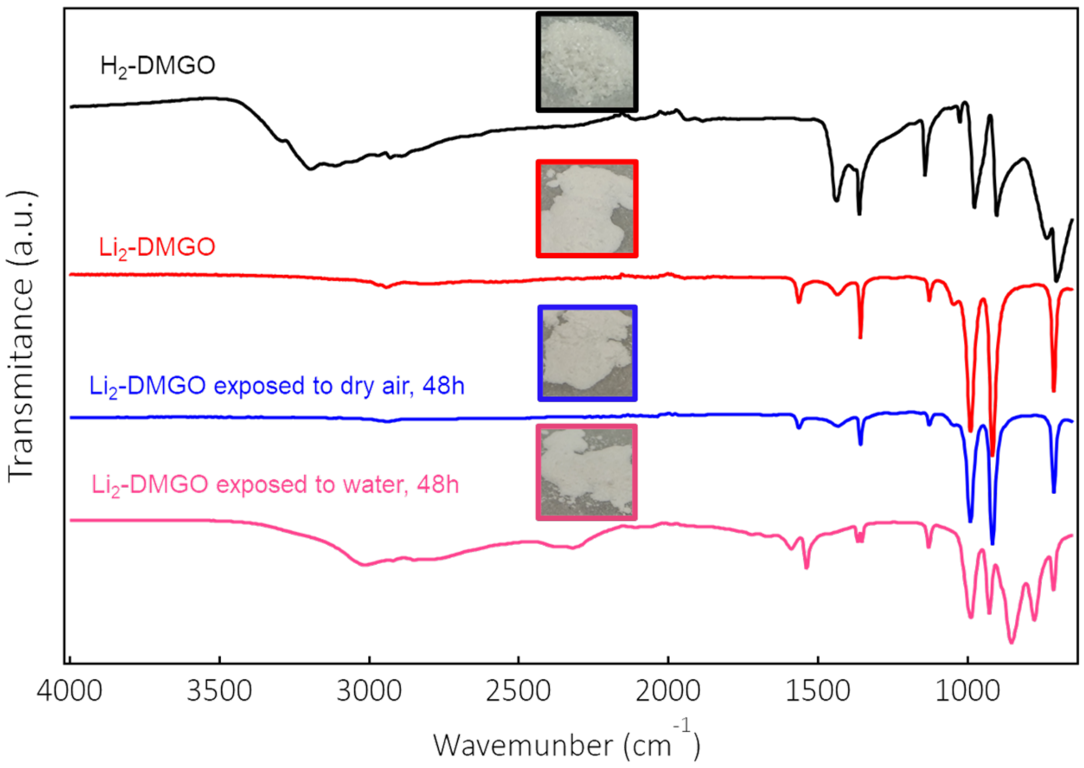

Supplementary Figure S16. FTIR analysis survey for the synthesis of Li_2-DMGO, coupled to air and moisture stability analysis.

The formation of lithiated product (Li_2-DMGO, red curve) is confirmed by the disappearance of the weak broad band between 3000-3400 cm^{-1} attributed to the hydroxyl groups of H_2-DMGO (black curve). Comparative FTIR spectra of pristine and dry air-exposed (for 48h) samples of Li_2-DMGO show identical FTIR signatures after dry air-exposure without any sign of decomposition (oxidation nor hydrolysis). For water vapor exposed Li_2-DMGO (pink curve), residual water or partial hydrolysis can be observed with the characteristic peaks still preserved after 48h of exposure.

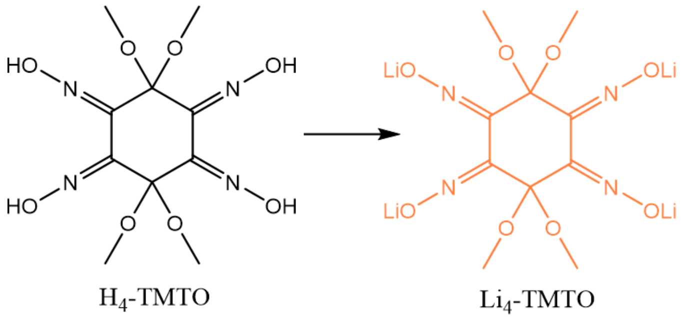

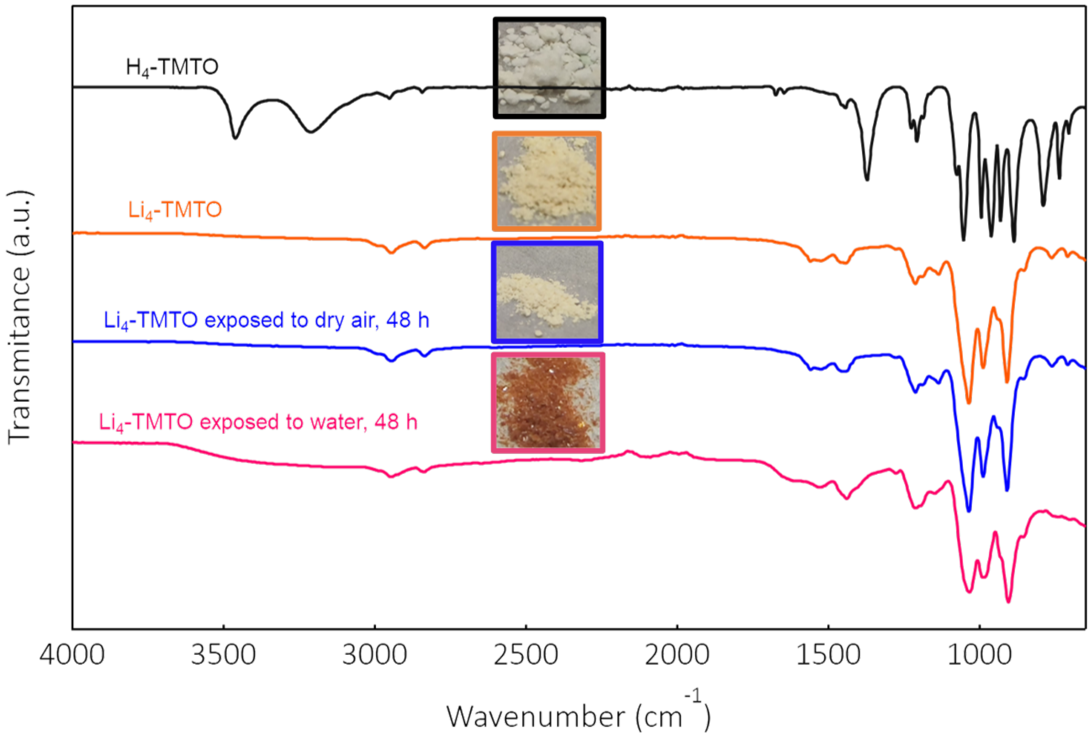

Supplementary Figure S17. FTIR analysis survey for the synthesis of Li_4-TMTO, coupled to air and moisture stability analysis.

The formation of lithiated product (Li_4-TMTO, orange curve) is confirmed by the disappearance of the two broad bands between 3000-3500 cm^{-1} attributed to the hydroxyl group of H_4-TMTO (black curve). Comparative FTIR spectra of pristine and dry air-exposed (for 48h) samples of Li_4-TMTO show identical FTIR signatures (blue curve) after dry air-exposure, without any sign of decomposition (oxidation nor hydrolysis). For water vapor exposed Li_2-DPGO (pink curve), residual water or partial hydrolysis can be observed with the characteristic peaks still preserved after 48h of exposure.

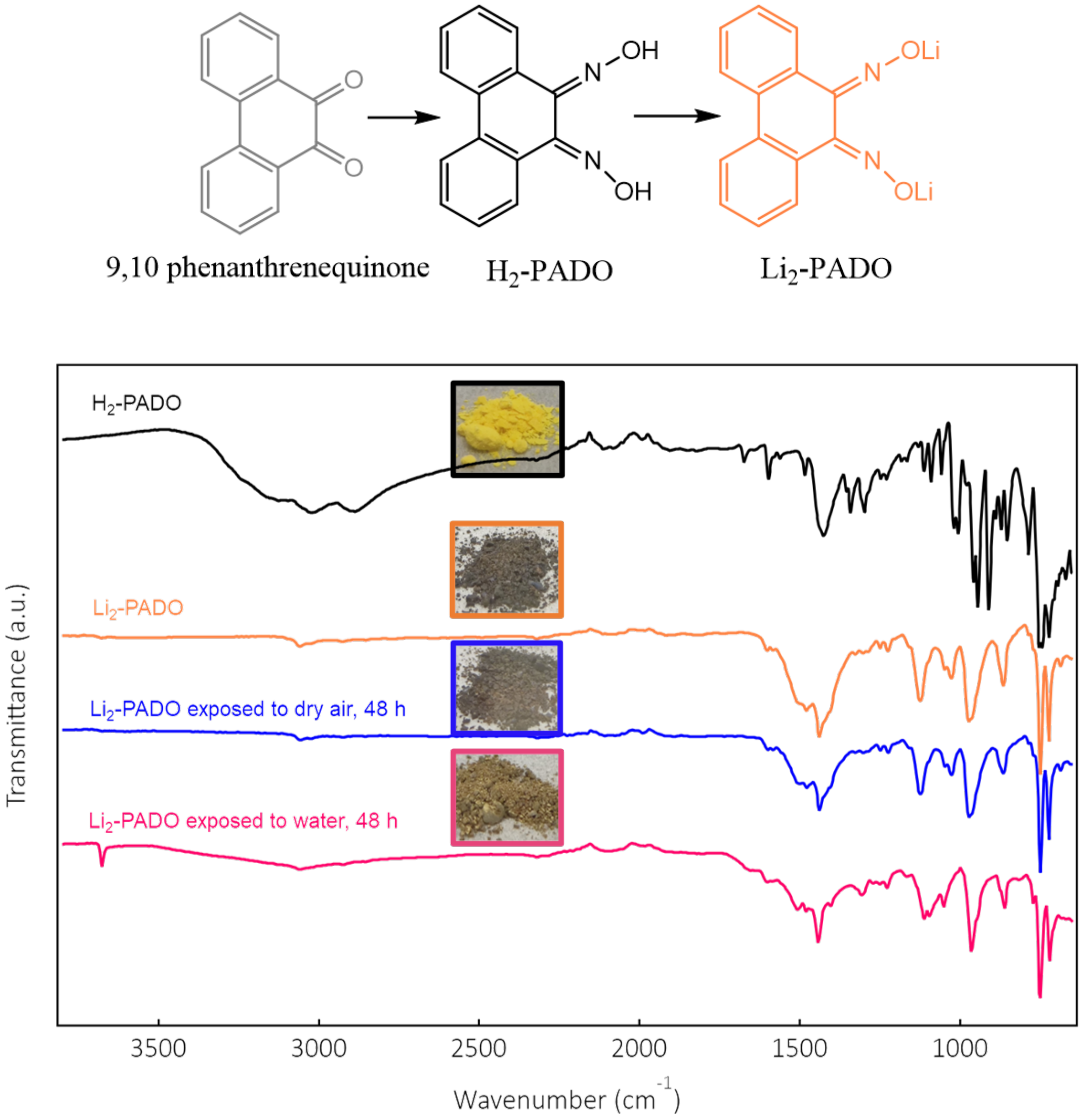

Supplementary Figure S18. FTIR analysis survey for the synthesis of Li_2-PADO, coupled to air and moisture stability analysis.

The formation of lithiated product (Li_2-PADO, orange curve) is confirmed by the disappearance of the broad band between 3000-3500 cm^{-1} attributed to the hydroxyl group of H_2-PADO (black curve). Comparative FTIR spectra of pristine and dry air-exposed (for 48h) samples of Li_2-PADO show identical FTIR signatures after dry air-exposure without any sign of decomposition (oxidation nor hydrolysis) proving stability under dry conditions as well as high oxidation potential (> 2.91 V vs. Li^+/Li). For water vapour exposed Li_2-PADO (pink curve), residual water or partial hydrolysis can be observed with the characteristic peaks still preserved after 48h of exposure.

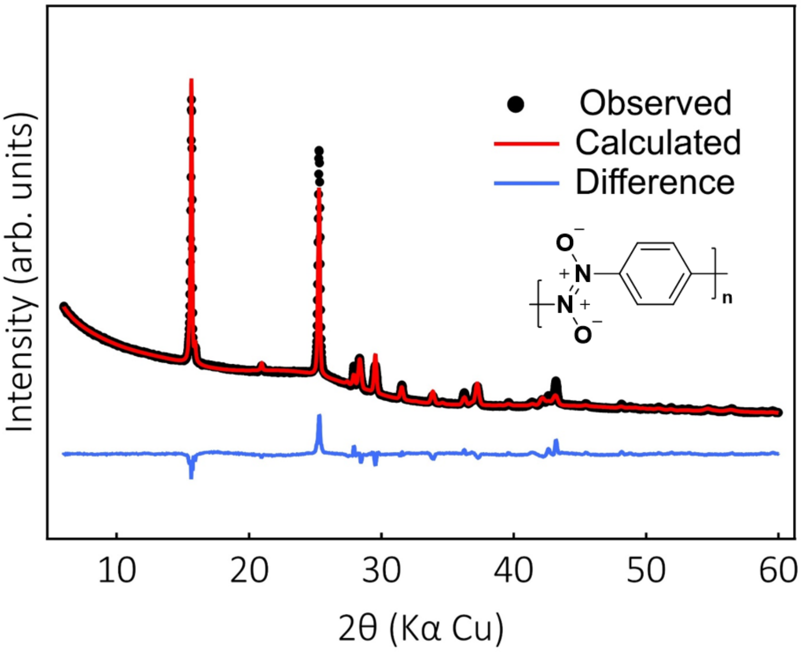

Supplementary Figure S19. Crystal structure analysis of PNND.

Experimental X-ray powder diffraction pattern (black dotted line) compared with the Rietveld-refined profile (red curve) and the difference (blue curve) for PNND.

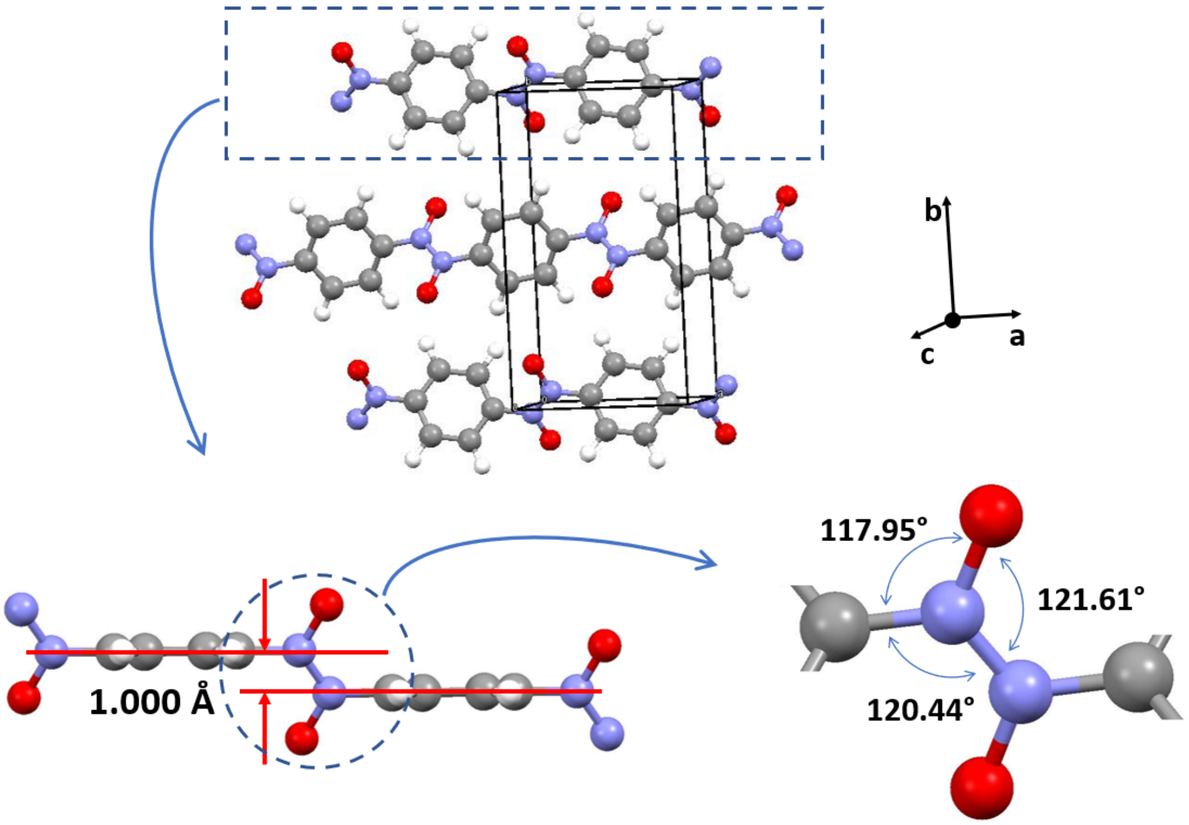

Supplementary Figure S20. Crystal structure of PNND polymer.

The crystal structure reveals that the polymer crystallizes in the monoclinic space group $P2_1/n$ (a = 6.394(2) Å, b = 11.322(4) Å, c = 3.7072(1) Å, β = 92.923(6)°, and V = 268.03(15) Å^3, Table S1). The structure is similar to the reported CCDC structure with reference no. 2006601. Crystal structure shows that the compound polymerizes following the *E* configuration along the N=N. Therefore, the PNND polymer is made up of linear chains, which go parallel along the crystallographic *a*-axis and stack along the *c*-axis. In the polymeric chain, the atoms do not lie on the same plane, but they are distributed on different planes defined by the aromatic ring and the -ON=NO- connection. The dihedral angle formed by the two planes leads to a stair-like structural motif in the polymeric chains.

Supplementary Table S6. Solubility of electrode materials in 1M LiTFSI in EC/DMC electrolyte formulation.

Entry	Composition	Solubility (mM)
1	Li_2-BQDO	5.25
2	PNND e-oxidized[a]	0.50
3	PNND chemically synthesized	0.18
4	Li_2-DMGO	6.42
5	Li_2-DPGO	6.40
6	DPODO	171.30
7	Li_2-PADO	4.21
8	Li_4-TMTO	1.77

[a] *Monomer molecular weight was used for molarity calculation. For calibration curve of chemically synthesized PNND, DMSO was used as a solvent. The analyzed sample was obtained by dissembling the cell with the Li_2-BQDO active material after 1st charge, followed by dispersing the composite mixture in 1M Li-TFSI in EC/DMC electrolyte. The resulting mixture was filtered through a 0.2 μ syringe filter and was further diluted 10 times with DMSO.*

For the neutral species (e.g. DPODO), high solubility was expected as the inter-molecular bonding in solid crystal are expected to be weak (no H-bonding, presumably mainly through VdW bonding) thus highly soluble in polar solvents.

For the PNND polymer, low solubility was expected, and also confirmed by measurements, while also keeping in mind the particular dissolution mechanism and species – the dissolution of PNND takes place via reversible dissociation of azodioxy dimers, generation of the dinitroso benzene intermediate, and solubilization of the later (as confirmed by GPC measurements).

The solubility of Li_2-BQDO, and of other ionic lithiated oximate species, was in turn found to be higher than expected. However, this expectation was not based on quantifiable or established rules, but merely on general knowledge with organic battery materials, as well as literature survey. Typically, the ionic organic compounds have low solubility in battery electrolyte polar solvents, this being one strategy set forward for organic battery material stable cycling (*3*). This can be assigned to relatively strong binding of Li-cations to the di-anionic organic center (or framework) and the solubility thus dependent on the solvation strength (polarity, dielectric constant) of the used solvent.

In the studied series of compounds, a trend can be indeed identified, with the di-anionic species (Li_2-BQDO, Li_2-DMGO, Li_2-DPGO, Li_2-PADO) having similar solubility in the range of 4-6 mM; whereas the tetra-anionic Li_4-TMTO has considerably lower solubility (< 2 mM) which could be explained by a stronger ionic binding to Li-ions, or dissolution via ion pairs to compensate for.

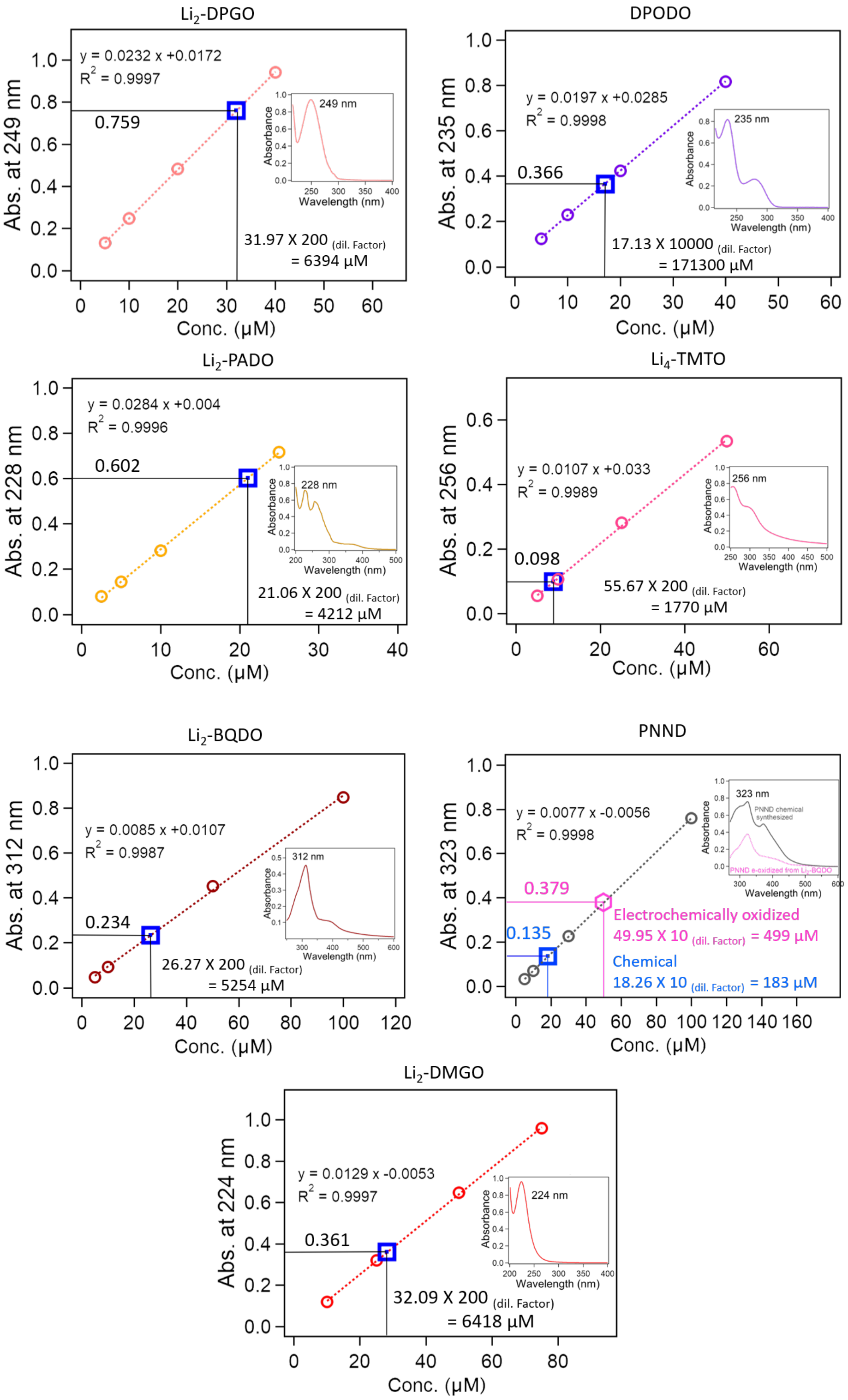

Supplementary Figure S21. UV-visible solubility calibration plots of the studied materials and their unknown diluted solutions in 1M Li-TFSI in EC/DMC electrolyte.

Except for PNND, stock solutions for all other electrode materials (Li_2-BQDO, Li_2-DMGO, Li_2-DPGO, DPODO, Li_2-PADO, Li_2-TMTO) were made in MeOH/ACN mixture, then dilutions were made with ACN to prepare the calibration curves.

Saturated solutions of each material were prepared in electrolyte 1M LiTFSI in EC/DMC electrolyte. The resulting mixture was filtered through Millipore Millex-HV PVDF 0.2 µm filters to remove the insolubilized solids. The filtrate was subsequently diluted in ACN/DMSO by a factor given indicated in every graph (Supplementary Figure S21, inset figure in each panel is respective UV-visible spectra.). Four different standard solutions were prepared for each material to make the calibration curves.

The calibration curves were obtained by linear fit of maximum absorbance of the standard solutions *vs* concentration using the Beer-Lambert law: A=εlC (A: absorbance; ε: molar extinction coefficient; l: length of the cell; C: electrode material concentration).

The solubility of the analysed materials was found to be relatively high in all cases (except PNND). These are following the trend (from highest to lowest soluble species): DPODO > Li_2-DMGO > Li_2-DPGO > Li_2-BQDO > Li_2-PADO > Li_4-TMTO > PNND e-oxidized.

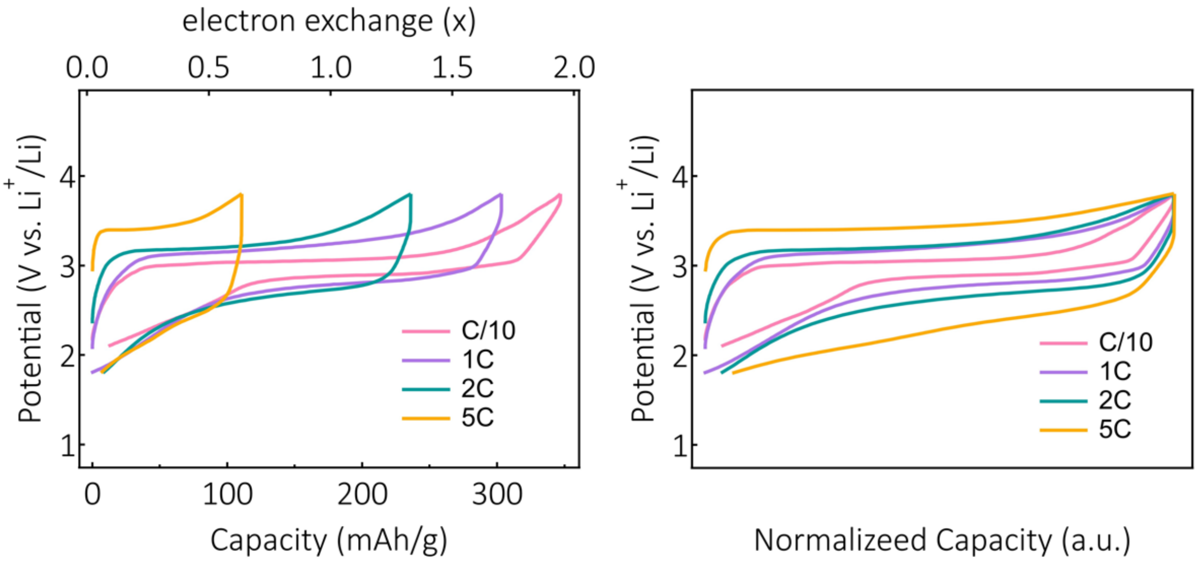

Supplementary Figure S22. Rate capability test of Li_2-BQDO.

The rate capability of the Li_2-BQDO electrode (active material mass loading of 4 mg/cm^2), measured at various C-rates, in a half-cell configuration, and in 7 M LiTFSI in EC/DMC used as electrolyte. The electrode can still reach a high capacity, close to 300 mAh/g, at a high 1C-rate (corresponding to 1 electron exchange in 30 min). From the Normalized capacity vs. Potential graph (right panel), an increase in the polarization with the increase of C-rate can be observed, attributed to a series of factors including working or Li-metal counter electrode polarization, as well as low ionic conductivity of the high concentration electrolyte.

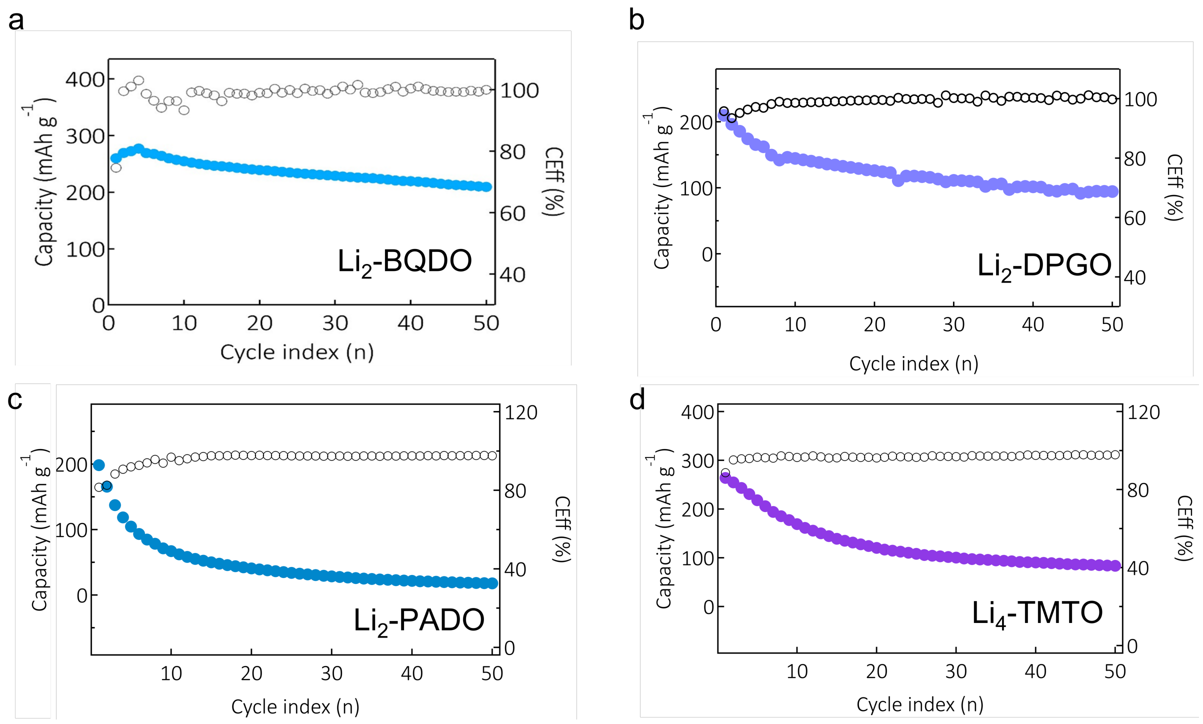

Supplementary Figure S23. Capacity retention and coulombic efficiency of selected oximates.

Cycling stability of cells containing Li_2-BQDO (a), Li_2-DPGO (b) and Li_2-PADO (c) as active material using a saturated LiTFSI in EC/DMC (1:1 vol%) electrolyte. The cell was cycled at a rate of C/2 (one electron exchange in 1h). Li_4-TMTO (d) in 1M LiTFSI EC/DMC (1:1 vol%) electrolyte with a cycle rate of C/10. Compared to dilithium-1,4-benzenediolate, which can barely be cycled for 5 cycles (*14*) (with more than 75% capacity loss) due to the very high solubility of 1,4-benzoquinone in electrolytes (*37*), Li_2-BQDO shows very stable cycling behavior with 75% capacity retention over 50 cycles. In contrast, Li_2-DPGO showed a moderate cycling efficiency of 49%, while Li_2-PADO exhibited poor performance with only 10% capacity retention, and Li_4-TMTO showed a 35% capacity retention after cycling. The superior cycling efficiency of Li_2-BQDO can be attributed to its low solubility compared to its homologous. The first cycle coulombic efficiency of Li_2-BQDO, Li_2-DPGO, Li_2-PADO, and Li_4-TMTO are of 76%, 95%, 79%, and 87%, respectively.

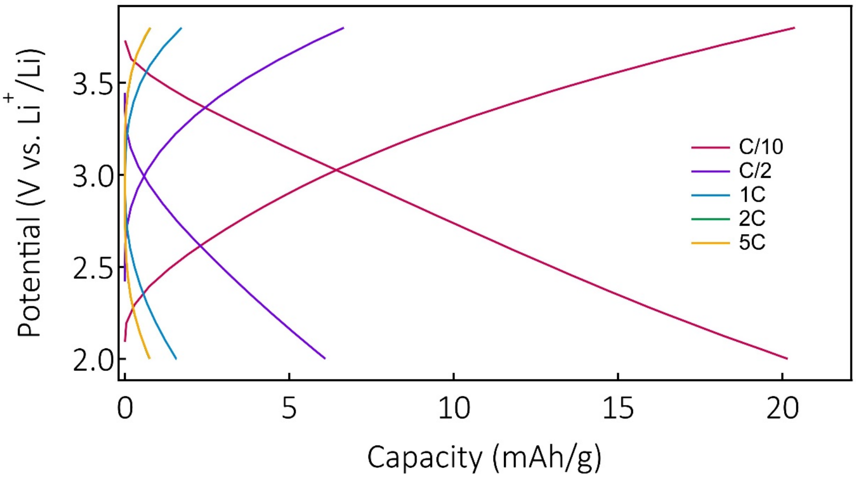

Supplementary Figure S24. Control experiment for the determination of the specific capacity contribution from Ketjen Black conductive carbon.

A pure Ketjen Black electrode was assembled in 2-electrode cell using Li-metal as counter/reference electrode and cycled in similar conditions as the studied active materials. For instance, the current corresponding to the C-rate applied in the graph above (for pure Ketjen Black electrode) is equal to the current densities applied to Li_2-BQDO electrode (Fig. S22).

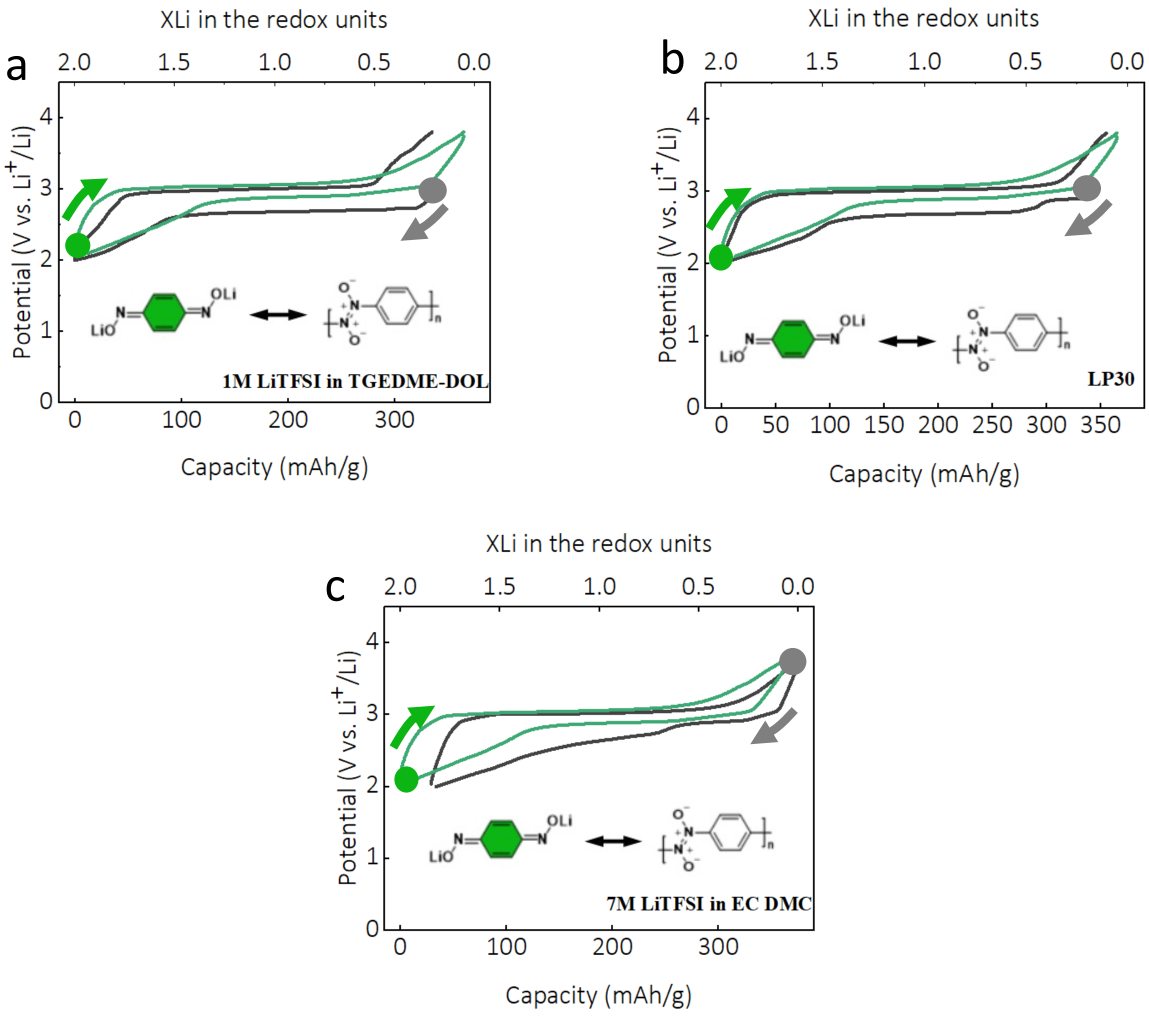

Supplementary Figure S25. Capacity-potential profile of chemically synthesized PNND and comparisons of cycling profiles in different electrolytes.

Comparisons of cycling profiles of chemically prepared PNND and Li_2-BQDO. The electrodes, cell assembly and testing were performed in similar conditions with the exception of the used electrolyte: (a) 1M LiTFSI in TEGDME-DOL; (b) 1M $LiPF_6$ in EC-DC; (c) 7M LiTFSI in EC-DMC. Overall, the chemically prepared PNND and Li_2-BQDO have similar galvanostatic cycling profiles, further corroborating the two-electron reversible redox mechanism between PNND (oxidized state) and Li_2-BQDO (reduced state), and the reverse process.

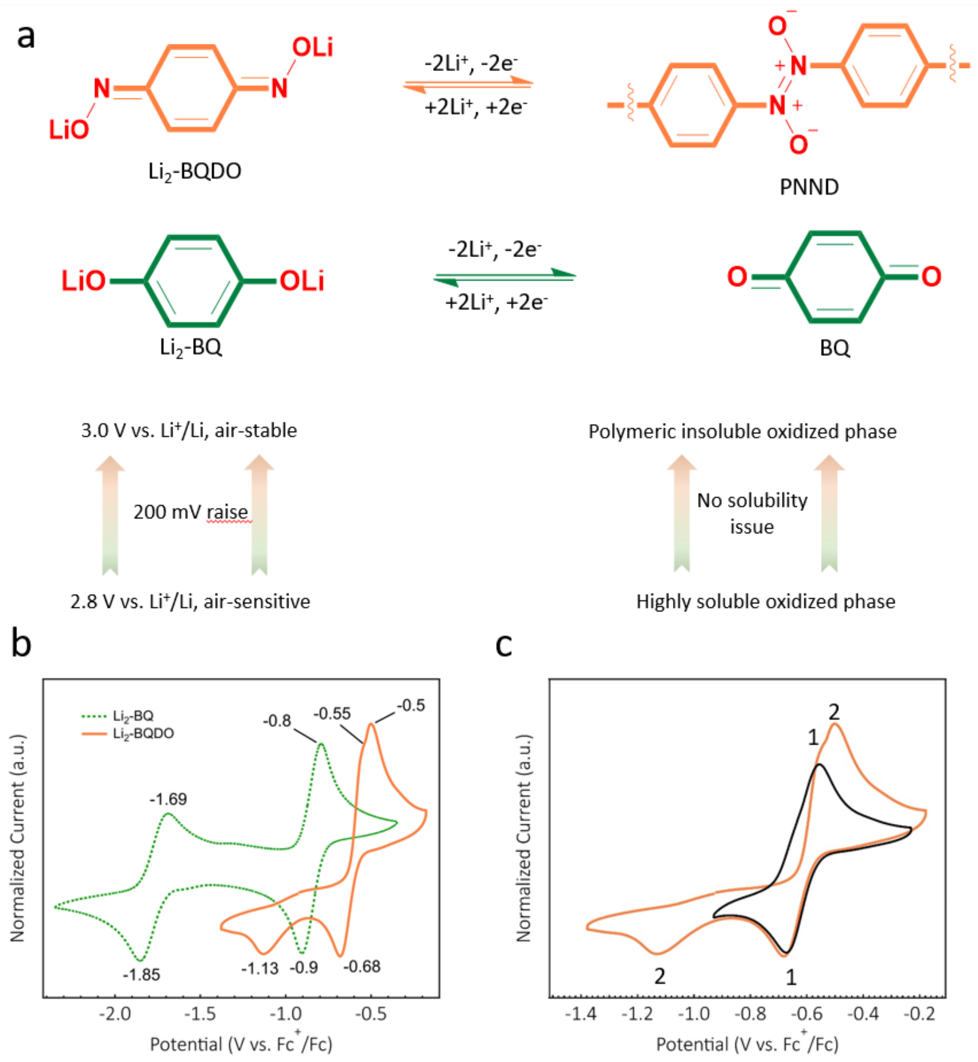

Supplementary Figure S26. Comparison of electrochemical properties for the main building units studied in this work: dilithium-1,4-benzenediolate (Li_2-BQ) vs. dilithium *p*-benzoquinone dioxime (Li_2-BQDO).

a) The redox reaction of Li_2-BQDO and of Li_2-BQ, highlighting the difference in the polymeric and single molecule oxidation reaction products. b) Cyclic voltammetry curve of Li_2-BQ 100 mM tetrabutylammonium perchlorate in DMSO) overlapped with CV of Li_2-BQDO (100 mM LiCl in DMSO). The measured potentials were calibrated with a Fc^+/Fc internal reference. The analysis shows higher redox potential of Li_2-BQDO compared to Li_2-BQ, with two pairs of redox waves (anodic peaks: -1.13/-0.55, -0.68/-0.5 V for Li_2-BQDO and -1.85/-1.69, -0.9 V/-0.8 V for Li_2-BQ vs. Fc^+/Fc), indicating a two-electron stepwise process. c) Cyclic voltammetry curves of Li_2-BQDO measured in two different potential windows: orange curve – full scan window, with the two cathodic processes observed, and narrow potential scan window (black curve) with only one pair of redox peaks noted. The redox pairs are noted with 1 and 2 and highlight the asymmetric anodic and cathodic reaction pathways of Li_2-BQDO.

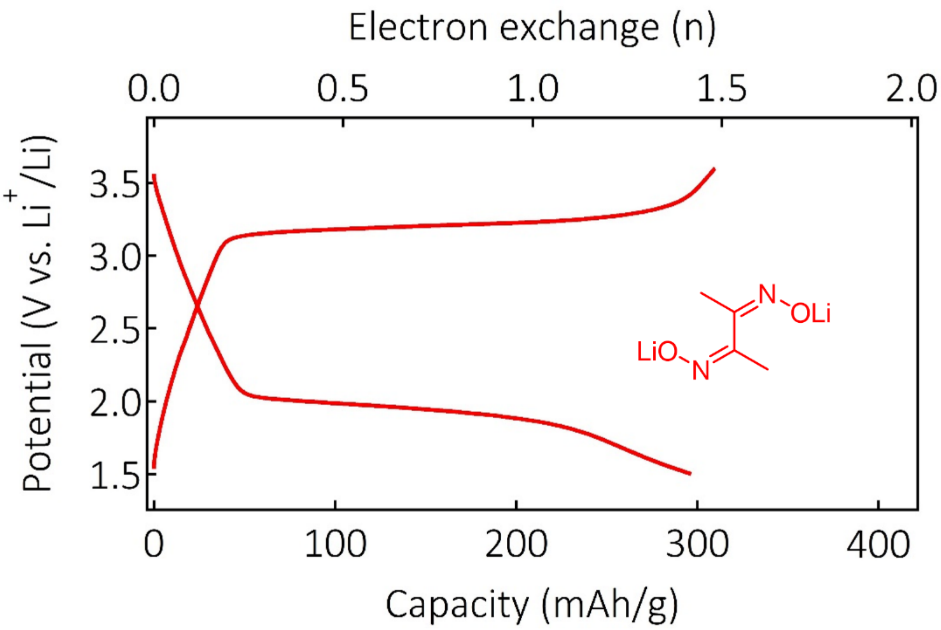

Supplementary Figure S27. Electrochemistry of dilithium-dimethylgyloxime (Li_2-DMGO).

Charge-discharge profile of Li_2-DMGO electrode material as measured in a Lithium half-cell. The measurement was done with cycling rate of C/10 in a 5 M LiTFSI in tetraglyme electrolyte, with an active material mass loading of 4 mg/cm^2. The charge plateau is located at around 3.1V vs. Li^+/Li, while the discharge plateau is around 2.1V vs. Li+/Li, resulting in a large polarization of nearly 1V. The material utilisation is of 1.5 electron exchange (for a theoretical of two electrons), explained by the high solubility and elution from the electrode of both Li_2-DMGO (Table S6) and its oxidized form (3,4-dimethyl-1,2,5-oxadiazole 2-oxide, that is a liquid) phases.

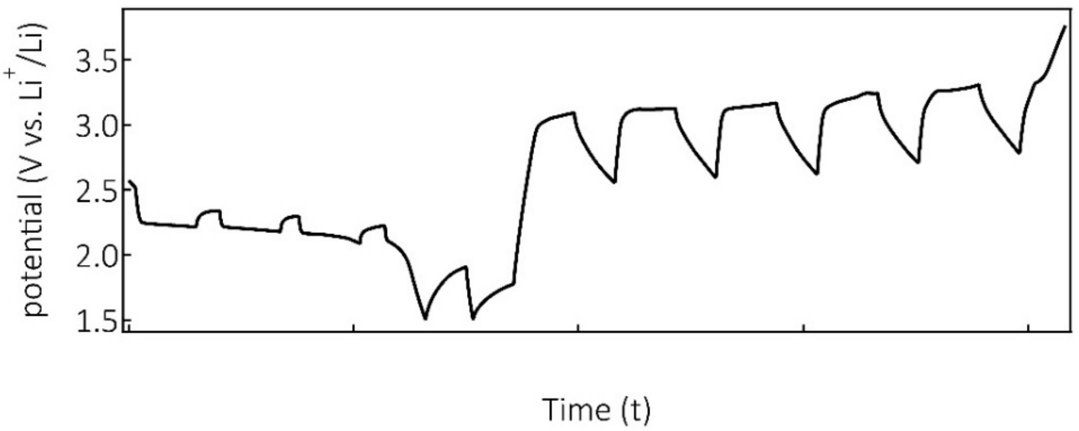

Supplementary Figure S28. Galvanostatic Intermittent Titration Technique (GITT) plot of the Li_2-DPGO – DPODO redox as measured in a Li half-cell configuration.

The test was performed with intermittent discharge/charge periods of 2 hours (rate of C/10) followed by relaxation periods of 1 hour. The data shows that the main contribution to the polarization originates during the oxidation process (Li_2-DPGO → DPODO, over 500 mV with the equilibrium not reached within one hour). Longer relaxation time have been also applied to allow the equilibrium to be reached, yet the solubility resulted in cells failure. The reduction process (DPODO → Li_2-DPGO) only contributes by ~150 mV to the overall polarization, with the equilibrium reached within 1 h of relaxation.

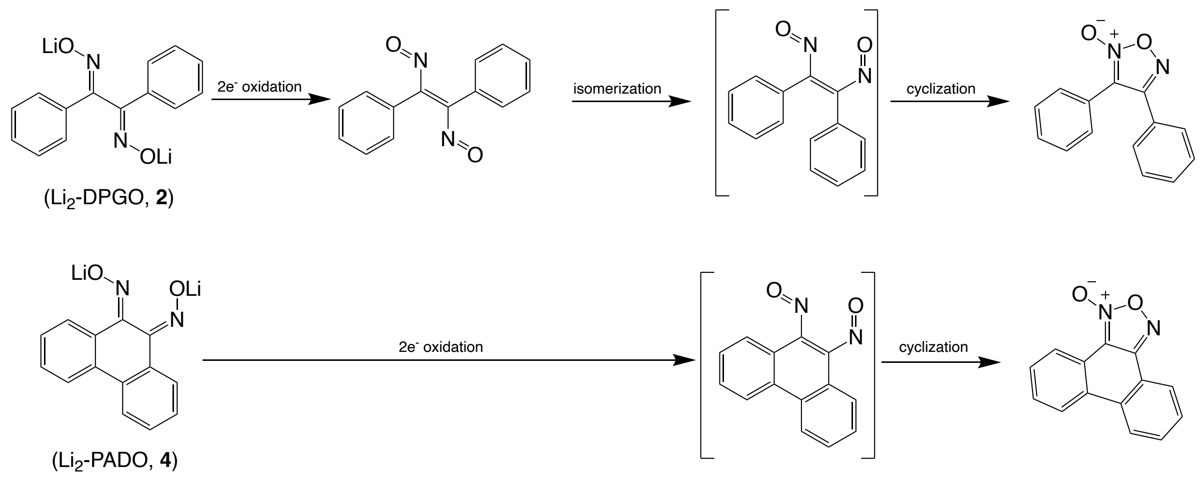

Supplementary Figure S29. Structural reorganization of Li_2-DPGO and Li_2-PADO during the oxidation process.

The aim of investigating the electrochemical performance of dilithium 9,10-phenanthrene dioximate (Li_2-PADO, **4**) was based on the assumption that the kinetically limiting step (and thus contributing to large polarization observed in galvanostatic cycling, Fig. 3 main text) is the *E-Z* isomerization step of (Li_2-DPGO, **2**). However, high large hysteresis of ~1V was also observed for Li_2-PADO, indicating that the *E-Z* isomerization is not the main contributor to the cell polarization (Fig. 3c). The experimental results have thus been corroborated to calculations for the energy profile of the Li_2-DPGO ⟷ DPODO conversion process (Fig. 3e, analysing both, linear and closed structure reaction pathways, and in both, gas and solid phases).

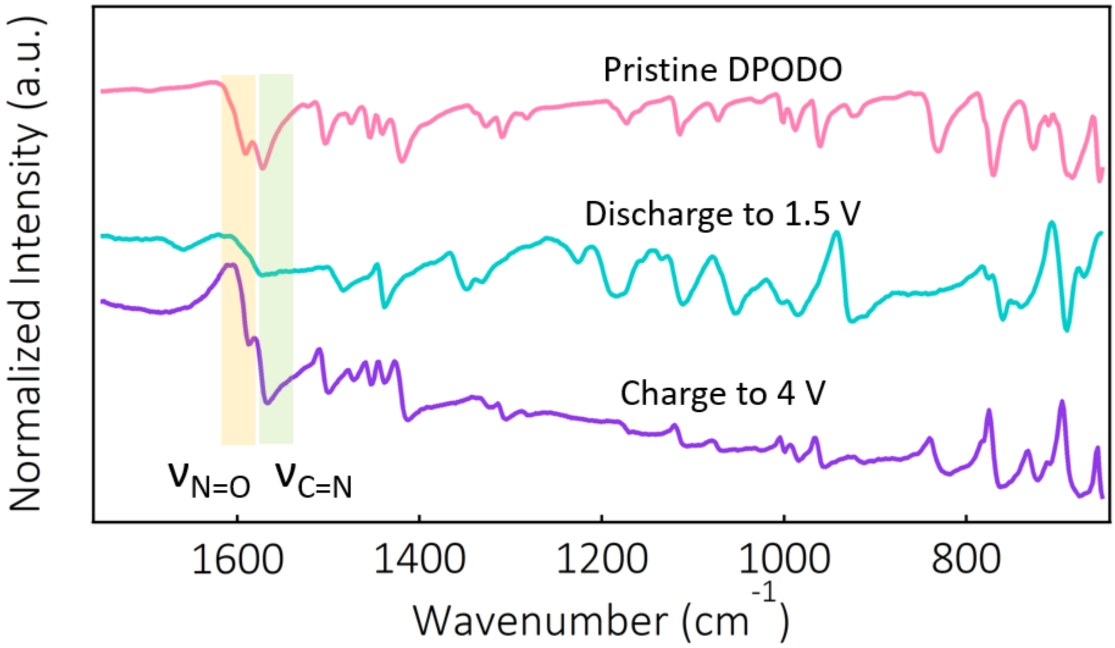

Supplementary Figure S30. Ex-situ FTIR survey of reversible electrochemical reaction between Li_2-DPGO and DPODO in solid phase.

Ex-situ FTIR analysis was carried out during the cycling of DPODO electrode (70% active material and 30% conductive carbon). Three different state-of-charge states were analysed: pristine (oxidized DPODO form), two electrons reduced (DPODO → Li_2-DPGO, discharged to 1.5 V vs Li^+/Li) form, and one full redox cycle (Charge to 4 V vs Li^+/Li, DPODO → Li_2-DPGO → DPODO). The analysis shows excellent reversibility, given the identical spectra between the pristine and full-cycled data. The full-discharged spectrum was also found similar to the one of the as synthesized Li_2-DPGO sample (Fig. S15), corroborating the electrochemical redox mechanism between DPODO and Li_2-DPGO.

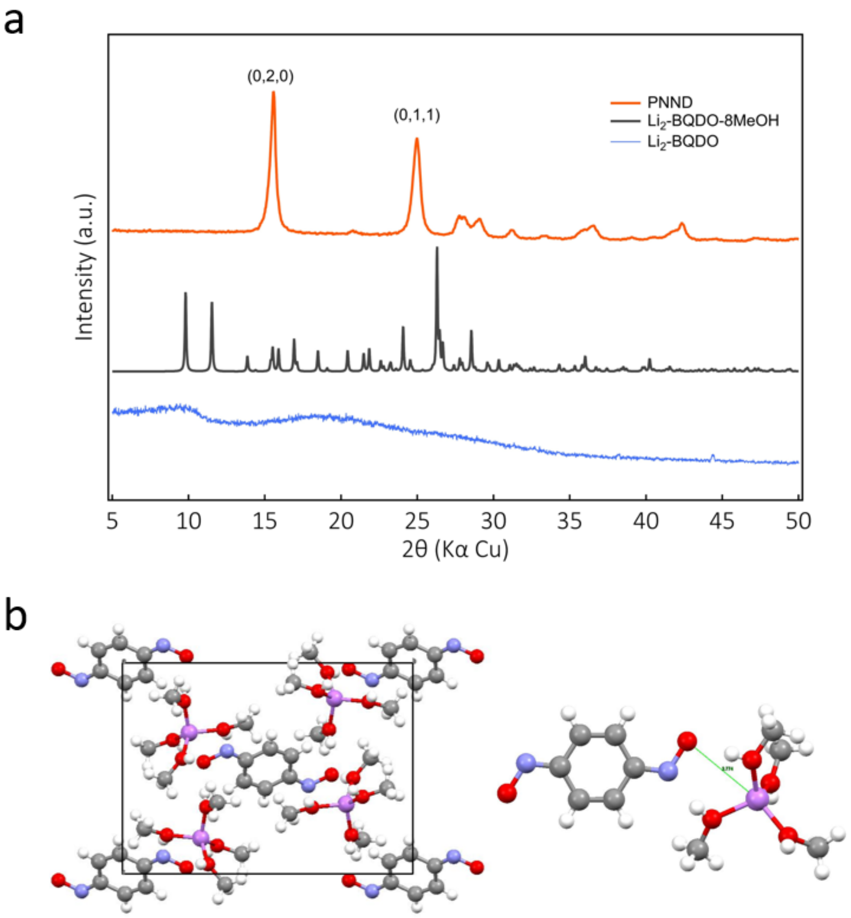

Supplementary Figure S31. PXRD data of Li_2-BQDO and PNND phases and analysis of Li_2-BQDO-8MeOH crystal structure.

(a) PXRD of PNND polymer, of Li_2-BQDO-8MeOH and of Li_2-BQDO phases. The crystal structure of PNND polymer was solved from the PXRD data (Figs. S19 and S20) whereas the crystal structure of Li_2-BQDO-8MeOH was obtained from single crystal data analysis. An interesting feature worth to be noted is that Li_2-BQDO-8MeOH is crystalline (black curve), whereas removing MeOH at 150 °C results in an amorphous Li_2-BQDO phase (blue curve). The amorphous nature of the Li_2-BQDO is preserved even during electrochemical charge-discharge cycles (Fig. 4, Main text), implying an exotic <amorphous small molecule salt> to <crystalline polymer> cyclic electrochemical conversion. (b) The single crystals of Li_2-BQDO-8MeOH were obtained by slow diffusion of diethylether into a solution of Li_2-BQDO in methanol at -30 °C over a period of one month. The X-ray diffraction analysis shows that Li_2-BQDO-8MeOH crystallizes in the monoclinic space group P 21/n (a = 7.0409(4) Å, b = 15.3351(10) Å, c = 11.1693(7) Å, β = 94.206 (6)°, and V = 1202.73 Å3. Each of the oximate function of the molecule binding two Li cations, which are solvated by four methanol molecules in a tetrahedral coordination.

Supplementary Table S7. Crystal data and structure refinement for Li_2-BQDO-8MeOH**.**

Empirical formula	C_{14} H_{36} Li_2 N_2 O_{10}
Formula weight	406.33
Temperature (K)	100(2)
Wavelength (Å)	1.54184
Crystal system	Monoclinic
Space group	*P*2₁/*n*
Unit cell dimensions (Å,°)	a = 7.0409(4)
	b = 15.3351(10)
	c = 11.1693(7)
	a= 90
	b= 94.206(6)
	g = 90
Volume (Å^3)	1202.73(13)
Z	2
Density (calculated) (g/cm^3)	1.122
Absorption coefficient (mm^{-1})	0.778
F(000)	440
Crystal size (mm3)	0.495x 0.453 x 0.329
Theta range for data collection (°)	6.935 to 67.096
Reflections collected	4644
Independent reflections	2109 [R(int) = 0.0311]
Completeness to q = 66.489° (%)	98.7
Absorption correction	Semi-empirical from equivalents
Max. and min. transmission	1.00000 and 0.86329
Refinement method	Full-matrix least-squares on F2
Data / restraints / parameters	2109 / 4 / 143
Goodness-of-fit on F2	1.083
Final R indices [I>2s(I)]	R_1 = 0.0402, wR_2 = 0.0992
R indices (all data)	R_1 = 0.0483, wR_2 = 0.1089
Dr (max,min)(e.Å-3)	0.182, -0.232

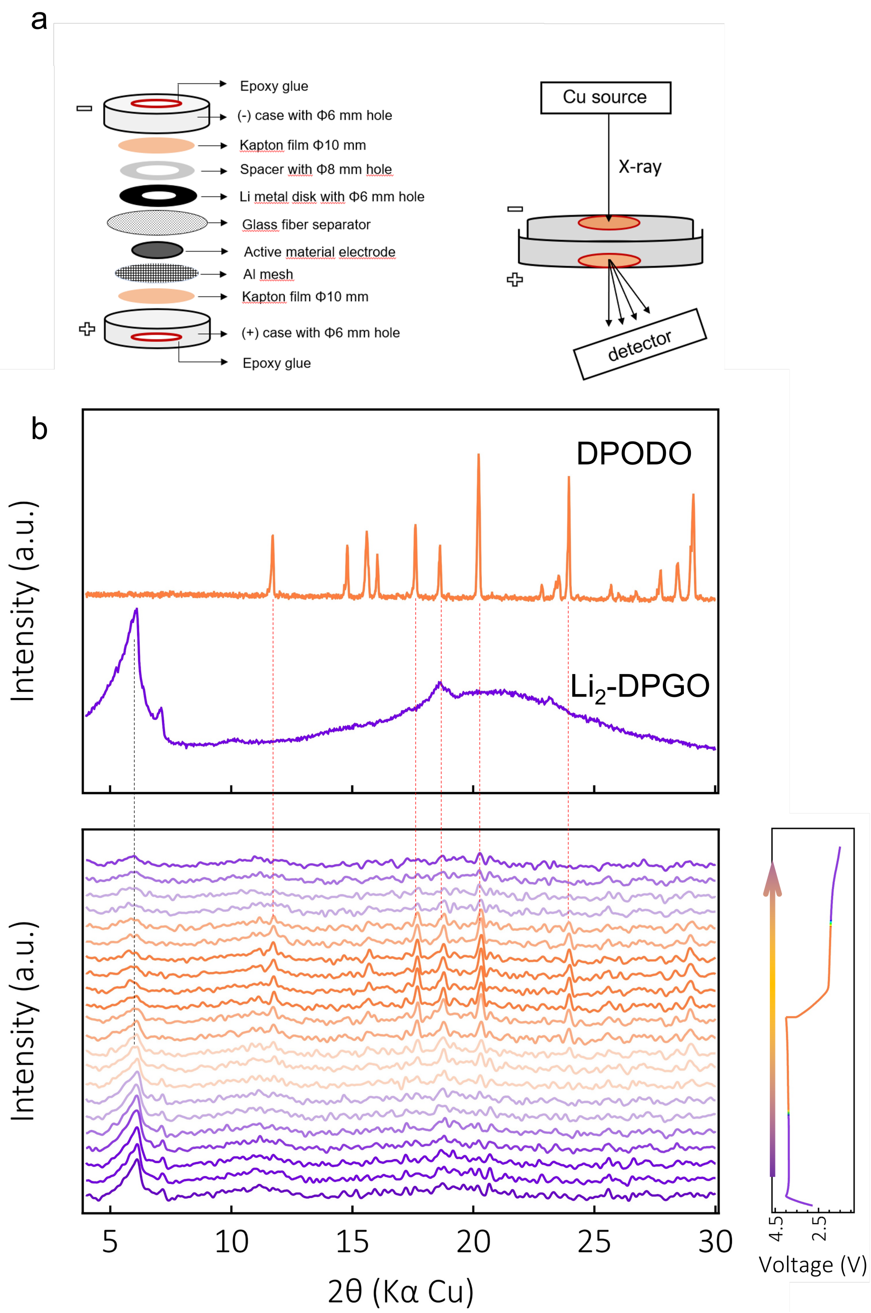

Supplementary Figure S32. In-situ XRD analyses. a) Schematic illustration of cell construct for the in-situ XRD measurements. b) In-situ XRD survey of Li_2-DPGO during electrochemical cycling in solid phase.

The as synthesized Li_2-DPGO is poorly crystalline (alike most of the Li-oximate derivatives disclosed in this work). Upon oxidation (charge), the Li_2-DPGO phase gradually vanishes, simultaneous to the appearance of a new phase assigned to DPODO (matching the PXRD pattern of the chemically synthesized single crystal of DPODO (*38*)). The forward and reverse reactions thus proceed through a simultaneous two-electron, bi-phasic mechanism, corroborating the formation of furoxan ring after oxidation of Li_2-DPGO (Fig. S29). Upon continuous cycling, the formed Li_2-DPGO phase becomes less crystalline.

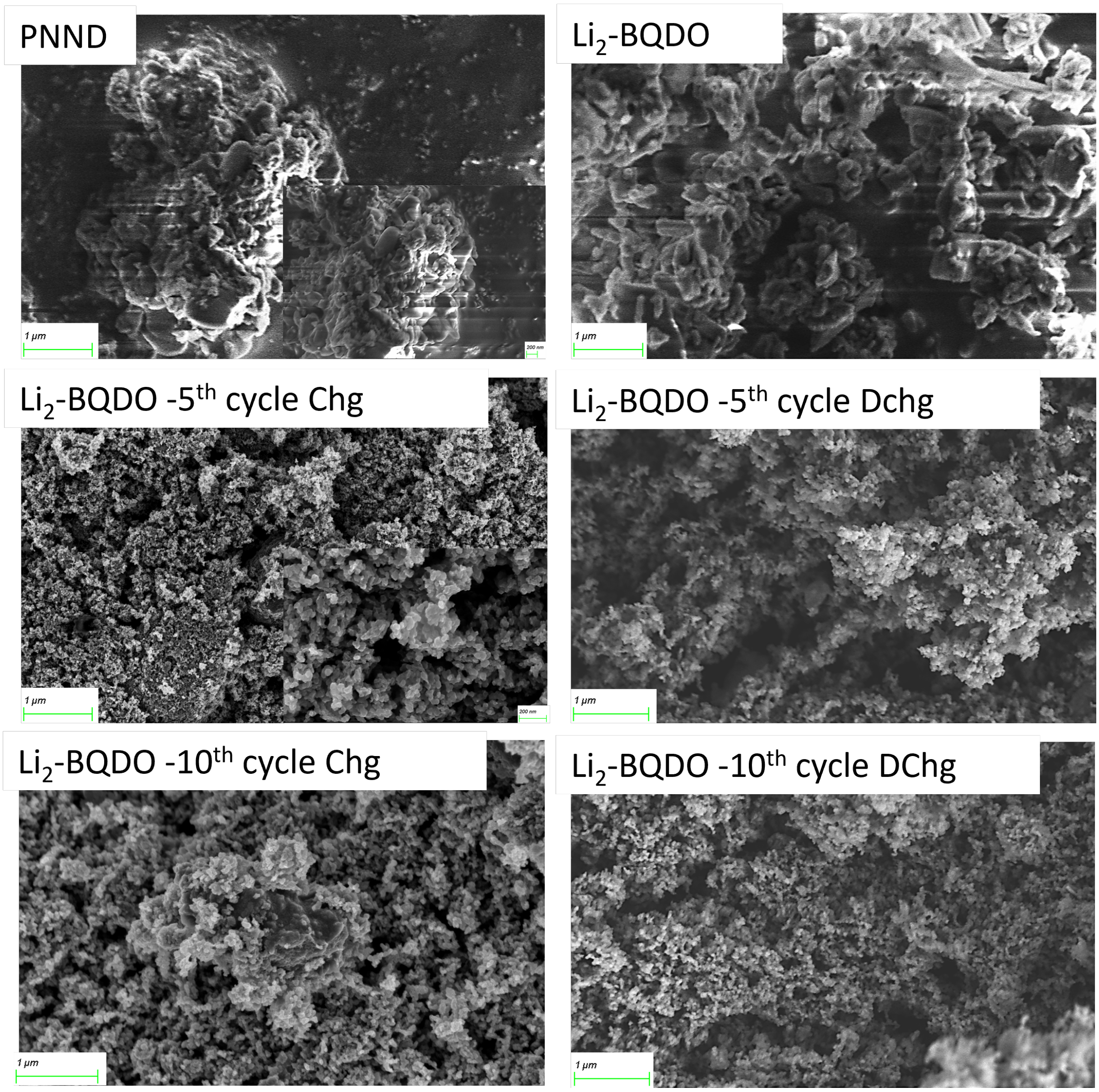

Supplementary Figure S33. Structural analysis of cycled electrodes.

SEM images of pristine PNND and Li_2BQDO electrodes as well as after different cycling sequences**.**

Supplementary Table S8. Electrical conductivity estimates of lithiated oximates.

Compound	σ (S/cm)
Li_2-BQDO	1×10^{-12}
Li_2-DPGO	5×10^{-13}
Li_2-DMGO	1×10^{-13}
Li_2-PADO	1×10^{-12}
Li_4-TMTO	2×10^{-12}

We conducted tests to measure the electronic conductivity at room temperature of the lithiated oximates described in this study. The tests were carried out on compressed powder samples (7 mm in diameter), placed between stainless steel rods and subjected to an applied pressure of 3T. We used two-probe d.c. current-voltage technique to measure conductivity. The estimates conductivities are in the 10^{-12} - 10^{-13} range, indicating insulating phases.

Gel Permeation Chromatography (GPC) analysis of soluble PNND species

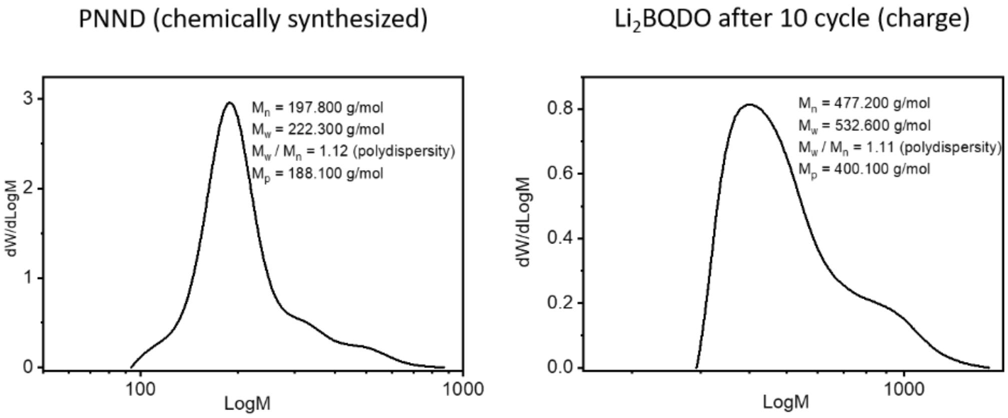

Figure S34. GPC analysis of chemically synthesized PNND (left panel) and electrochemically synthesized PNND (right panel).

The electrochemically synthesized PNND was prepared by galvanostatic cycling (10 full cycles) of Li_2-BQDO, stopping the cycling in fully charged (oxidized) state, disassembling the cell, washing the positive electrode with DMC, followed by drying.

The PNND has low solubility in common solvents. Only partial solubility was observed in DMF and DMSO (Table S6) after long sonication. After this step, the DMF dispersion was centrifuged and the supernatant was filtered through a 0.2-micron filter before GPC analysis. The GPC was performed on an Agilent gel permeation chromatography (GPC) system equipped with an Agilent 1100/1200 pump (25°C; eluent: DMF, 2.5mM of NH_4PF_6; flow rate: 1 mL/min). The estimated M are relative masses since these are determined with respect to polystyrene standards which have different hydrodynamic and elution volumes as compared to PNND, which is primarily composed of monomers and small-chain oligomers.

The analysis shows that the soluble species are of low M, in the range of the molecular weight of the 1,4-dinitrosobenzene monomer (136,11 g/mol), with a series of peaks with lower intensity and corresponding to integer multiples of the main peak M_W. The low polydispersity (1.11 – 1.12) for a potentially non-controlled polymerization process is also an indication that the species resulting from the solubilization of PNND are monomers or a few units (2-5) oligomers.

The solubilization via depolymerization can be explained by the low activation energy for the dissociation of azodoxy dimers (20–30 kcal/mol), implying that this covalent bonding can be broken – in this case, under the influence of polar solvents. This is also supported by previous findings wherein mono-nitroso compounds have been observed to behave differently in dissolved and solid phases: faintly yellow in the solid state, bright green in solutions (*31*). This was attributed to the reversible dimerization of nitroso compounds with most nitrosoarenes thus existing as (dissolved) monomers in solution, and dimeric in the solid state, and at room temperature.

DFT Calculation Section.

The case study of Li_2-DPGO ⇄ DPODO conversion.

1 Computational setup.

DFT was performed for both gas- and solid-phase systems. The gas-phase simulations were performed with the GAMESS package (*56*). Total energies for each of the oxidation states, E_{tot} were computed for the relaxed structures using the Minessota M11/M15 exchange-correlation functionals (*57*) and the 6-31G** Pople basis set. Solid-state calculations were performed using SIESTA (*58*) which uses norm-conserving pseudopotentials and LCAO representation of the wavefunction. The exchange correlation functional used was PBE for solid (PBEsol (*59*)); this choice being known to produce reliable geometric structures for molecular crystals bulk states (*60*). In order to take into account the van der Waals interaction, we applied the Grimme's corrections to PBEsol (*61*).

Geometric models.

Gas phase calculations – were performed for two structures: closed and linear one (Figure S35). In all cases, the results are reported after performing a structural relaxation up to a gradient of 0.01 eV/Å.

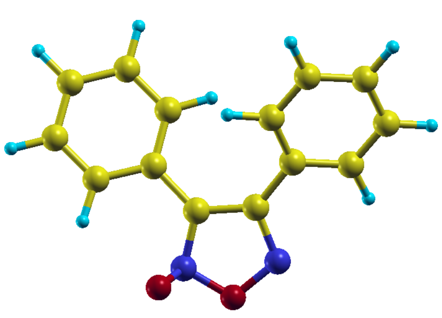
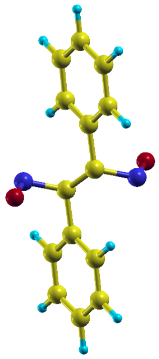

Supplementary Figure S35. Ball-and-stick representation for closed (left) and linear (right) structures of DPODO and $DPGO^{2-}$ in gas phase.

For the solid-phase calculations we start out investigations for two types of packing (i.e. for closed and linear structures, respectively). We used two types of computational models: in the first one, the structural relaxation is applied only to atoms in the cell (experimental geometry of the cell is preserved); second, the structural relaxation of the atomic positions and cell parameters is performed. For all models the structural relaxation was conducted to attain a gradient of less than 0.05 ev/Å. Results for the relaxed cells are given in Table S7.

Supplementary Table S9. Experimental (bold) and calculated values for the crystal structures for closed (Row 1) and for the linear structures (Row 2).

	***a* [Å]**	***b*[Å]**	***C* [Å]**	**Alpha (°)**	**Beta (°)**	**Gamma (°)**
**closed**	15.15/14.28	12.26/12.25	12.66/11.64	90/89.95	94.36/101.05	90/89.97
**linear**	11.42/11.95	20.01/20.43	11.70/8.90	90/90	114.71/115.01	90/90

To investigate the insertion of Li in the structures we used supercell models with 2×2×2 unit cells, allowing more freedom to Li-ions (i.e. with respect to periodic boundary conditions) for both close/linear structures.

We calculated three models corresponding to a gradual insertion of Li-ions into the organic bulk: no Li atoms included, 50% of the maximum amount of Li atoms (i.e. 8 atoms in each super-cell i.e. a single Li atoms per molecule) and 100% of Li atoms (i.e. 16 Li atoms per supercell i.e. 2 Li atoms per molecule). The initial guess for the positions of Li atoms was produced by generating random positions at distances between 2.8 and 2.5 Å around the oxygen atoms, followed by full relaxation up to 0.05 ev/Å. As an example, the relaxed structures for 16 Li atoms in the two types of supercells in given in Figure S35.

The effect of Li insertion (i.e. 100% of Li atoms in the structure, that is 16 atoms/supercell) upon the cell parameters is presented in Table S9. As a qualitative comment we note that the effect of Li insertion upon the volume change of the unit cells is opposite for the two structures: for closed structure the volume is increasing in presence of Li, while for the linear one the volume is decreasing.

Supplementary Table S10. Effect of the Li insertion upon the structural parameters of the crystals: calculated values for the crystal structure parameters in presence of Li atoms for closed structure (Row 1, volume = 2050.03 Å^3 compared to 2344.65 Å^3 without Li) and for the linear one (Row 2, volume = 2014.04 Å^3 compared to 2427.212 Å^3 without Li). For comparison, refer to the values in Table S7 for the structures without Li.

	***a* [Å]**	***b*[Å]**	***C* [Å]**	**Alpha (°)**	**Beta (°)**	**Gamma (°)**
**closed**	15.21	10.53	12.86	87.16	84.76	89.38
**linear**	10.25	17.25	11.78	90.52	104.93	89.52

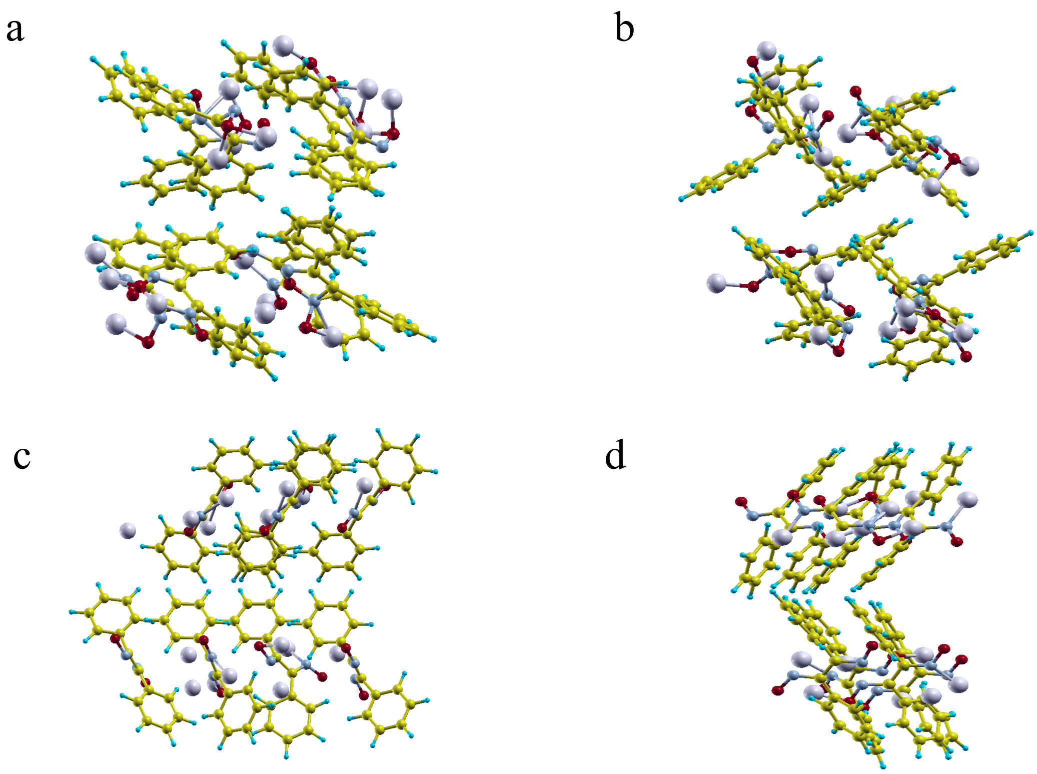

Supplementary Figure S36. Representation of the supercells used in calculations for closed structure (a and b) and for the linear one (c and d). Maximum number of Li atoms (i.e. 16) - represented with large gray balls – is included in each structure.

2 Results and Discussions.

2.1 Geometric structures.

The statistical distribution of selected bond lengths in various states was monitored by using histograms of interatomic pair distances. Since the system is periodic, not all the pairs are properly considered (i.e. the bonds at the border of the periodically repeated cell); the analysis on larger super-cells was thus performed, and then re-normalizing the total number to the cell used in calculations. Non-integer numbers in the histogram indicate atoms that are not properly taken into account due to periodic boundary conditions.

- → **Li-O and Li-N pairs:** A total of 100 steps between 1 Å and 5 Å was used to build the histograms, with the results for closed and linear structures being given in Figures S36 and S37, respectively. It can be noticed that for closed structure (Figure S36), Li atoms have the tendency to coordinate O atoms with a majority of Li-O at a distance of about 1.9 – 2.0 Å. For the linear structure (Figure S37), Li atoms are coordinated equally to O and N atoms, with a slightly higher number of Li-N pairs (around 40), at distances equal to 2.1 and 2.3 Å, respectively, and similar number for Li-O pairs at distances of around 1.8 – 1.9 Å.

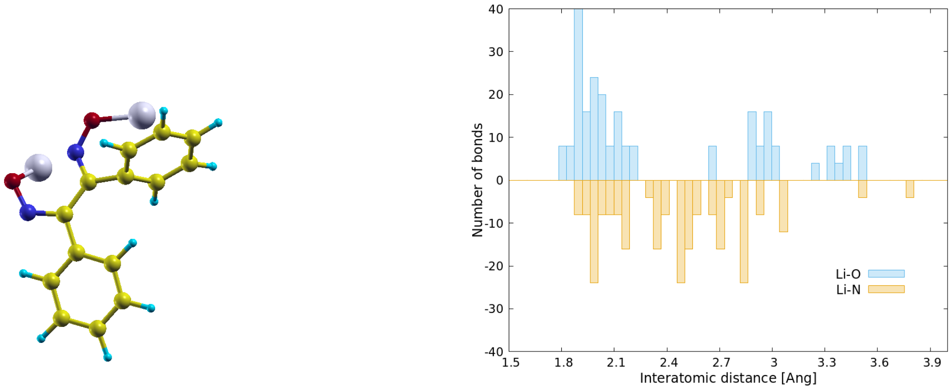

Supplementary Figure S37. Geometric analysis for closed structure. *Left*: geometric structure of a selected molecular unit in the bulk after structural relaxation in the presence of Li atoms (gray balls) – showing that the closed structure is not preserved in presence of the two Li atoms (i.e. the N-O bond is broken). *Right*: histogram count of the Li-O (blue) and Li-N (yellow) distances in presence of Li atoms. While for the Li-N we note a more disperse distribution of the bonds, the values of Li-O are grouped around value 1.9 Å. Large distances (e.g. over 2.5 Å) are purely geometric results, no chemical interactions can be present.

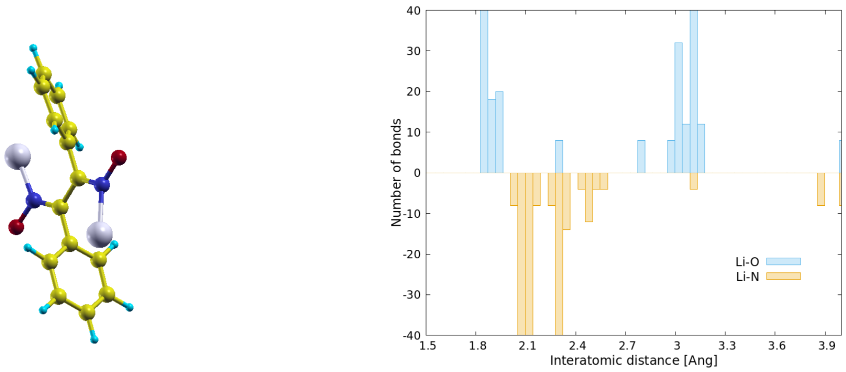

Supplementary Figure S38. Geometric analysis for linear structure. *Left*: geometric structure of a molecule in the bulk after structural relaxation in presence of Li (gray atoms). *Right*: histogram count the Li-O (blue) and Li-N distances (yellow), respectively, in presence of Li atoms; the distances are grouped around 1.9 Å (for Li-O), and 2.1 - 2.3 Å (for Li-N).

• → **C=N bond:** To investigate the C=N bond evolution upon redox conversion, a smaller step in the histogram (0.002 Å) was used, since the C=N is a significantly stronger bond. The results for the two structures and for the three Li concentrations (i.e. 0%, 50% and 100%) are presented in Figure S38. In the absence of Li (no distortions of the structure) the number of corresponding C=N bonds in the histogram is obtained. For the linear structure (top), in absence of Li, sixteen C=N bonds with lengths around 1.344 Å (i.e. all bonds are equal in the absence of Li) are counted. For the closed structure (bottom), two groups of eight C=N bonds, with lengths around 1.342 Å and 1.352 Å, respectively, are noted – corresponding to the two types of C=N bonds in the closed structure.

The presence of 50% of all Li atoms leads to a random distribution of the C=N bonds for both structures. Indeed, the orange histogram indicates an almost uniform distribution of the bonds, which is the indication that no dominant structure is present in the two structures (linear or closed one).

For the fully saturated structure (100% Li), we note that in the closed structure the presence of a first Li atom has the effect that all C – N distances tend to increase: all values are superior to 1.34 Å (i.e. the bond length in absence of Li), with about ten bonds counted for 1.36 Å. For the C=N bond, this represents a weakening of the chemical bond in presence of Li atoms (longer distance, weaker bond). For the linear structure the effect is opposite, with many bonds centred around 1.33 Å (inferior to 1.345 Å in absence of Li). This indicates stronger bonding in presence of Li for the linear structure.

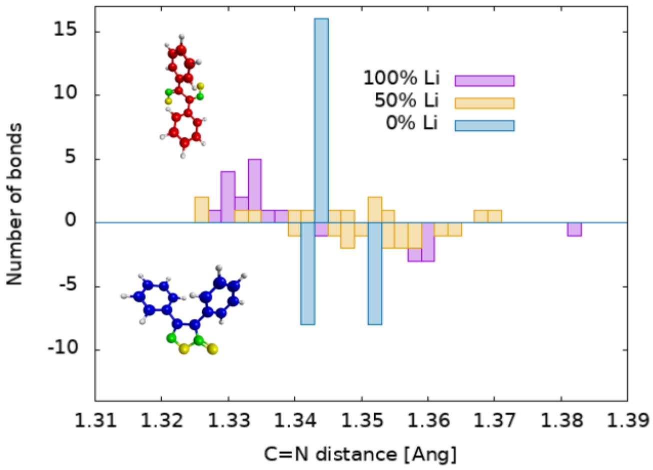

Supplementary Figure S39. Histogram of the C-N distances (nitrogen represented with green in the inset of the pictures, carbon with blue) as function of Li concentration for the two structural models presented in Figure S35. Top values– linear structure; bottom values – closed structure. It can be noted that presence of Li (yellow and purple boxes) has an opposite effect on the C-N bond length in the two structures: smaller (1.333 Å) for linear configuration, and larger (1.360 Å) for the closed configuration.

Summary of the geometric properties analysis:

The impact of Li atoms on the molecular configuration for the two structures in solid phase (i.e. closed and linear) has an opposite effect, as follows:

- *The volume of the elementary cell*: linear structure has a lower volume of the unit cell, while the closed one has a larger volume of the unit cell, upon the insertion of Li.
- *Strength of C=N bond*: in the linear structure a shortening of the bond is brought by the presence of Li, while for the closed configuration, the bonds are longer (i.e. weaker).
- To note that the weakening of the C=N bond in closed structure leads to a complete break-up, so that the structure is not stable in presence of Li.
- Finally, the statistical analysis of the Li-O / Li-N distances reveals that Li is coordinated predominantly to O in the closed configuration, while for the linear one a similar statistical trend of Li-O and Li-N bonds is observed.

2.2 Energetic stability.

The total energy of the systems in bulk state and as free molecules, for each oxidation state was calculated. Energetic diagram in gas-phase of the closed/linear structures is represented in Figure S39, for relaxed molecules bearing total charges 0, -1 and -2. The calculations show that the closed structure is not stable for -2 charge (i.e., after structural relaxation at -2 charge, the closed *structure is transformed into the linear one*). It can be concluded that in gas phase, the most stable structure is the structure with total charge Q=-1, while the presence of the second electron is destabilizing the structure.

The diagram for total energy of the bulk states with different amounts of Li atoms inserted in the structure (i.e. 0 %, 50% and 100% of the total 16 atom/supercell) is given in Figure S40. We note that the presence of Li leads to an energetic stabilization of the structures. If no Li atoms are present, the closed structure is more stable, while in presence of 100% Li atoms the linear one is more stable. In the intermediate case (only half of Li atoms are insert) we found structures with energies that are close to each other (around 0.5 eV difference).

To be noted that the results on energetic stability corroborate those on the volume cell calculations in presence of Li. Indeed, for the linear structure we found that in presence of Li the cell volume diminishes, indicating a more efficient packing, while the total energy of the system shows an important energetic stabilization in this case.

T*he overall energy difference per Li atom* (difference between the most stable structure in the two redox states / molecule / Li atom) is 0.67/0.72 eV for models with experimental / relaxed cell parameters, respectively.

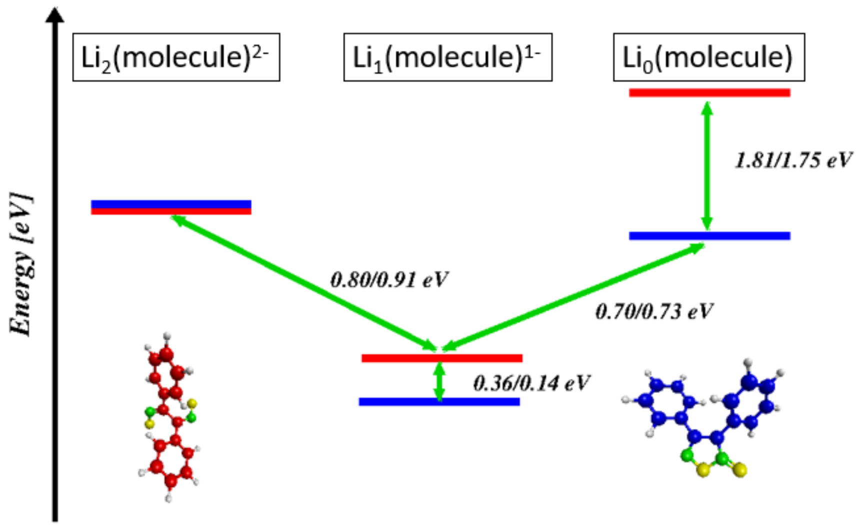

Supplementary Figure S40. Diagram of total energy/molecule for various oxidation states (total charge: 0, -1 and -2 corresponding to Li_0(molecule)=[DPODO], Li_1(molecule)$^{1-}$=[Li_1-‘DPGO’] and Li_2(molecule)$^{2-}$=[Li_2-DPGO], respectively) for linear/closed structures (represented with red/blue lines) in gas phase. The differences (expressed in eV) are indicated for two types of exchange-correlation functionals, M11 and M15, respectively.

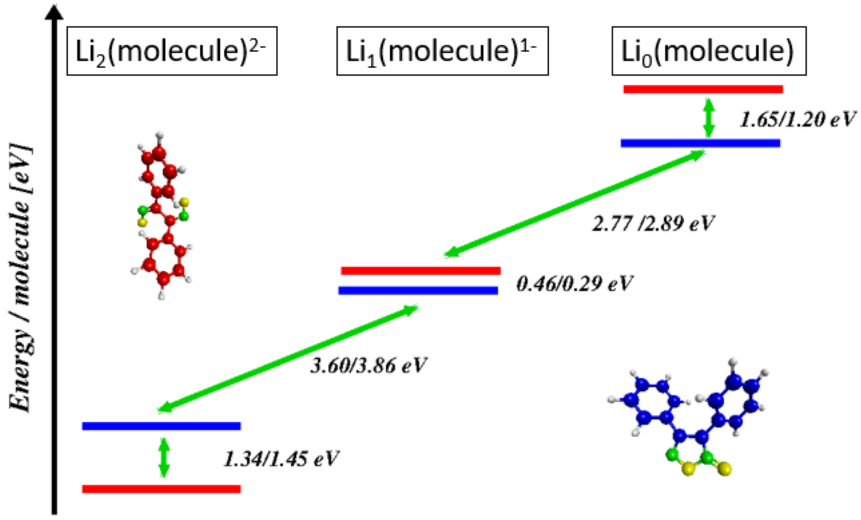

Supplementary Figure S41. Diagram of the total energy/unit cell (i.e. 8 molecules) for various oxidation states (amount of Li per molecule formulation: Li_0(molecule)=[DPODO], Li_1(molecule)$^{1-}$=[Li_1-‘DPGO’] and Li_2(molecule)$^{2-}$=[Li_2-DPGO]) for linear/closed structures (represented with red/blue lines) in bulk phase. The differences (expressed in eV) are indicated for two types of calculations: based on the experimental cell parameters / calculated cell parameters (see Tables S9 and S10), while atomic positions are fully relaxed in all cases.

2.3 Summary on the energetic properties:

- Gas phase calculations indicate that a charge -2 leads to an energetic destabilization of the molecule. The closed structure does not exist in this state (transforms into a distorted linear configuration).
- In bulk form both types of structures (closed and linear) can accommodate one or two Li atoms per molecule and this is increasing their energetic stability. However, we mention that the molecular units of the closed structure are transformed to the linear structure in presence of Li atoms (i.e. similar to gas phase).
- Main difference between gas phase and bulk structure is that in bulk the presence of Li (i.e. ionized molecules) leads the most energetically stable structure, while for gas the molecule with charge -2 represents the less stable structure.
- We conclude that this is entirely a consequence of intermolecular interactions in bulk phase. Consequently, the redox potential should be depicted as strongly dependent on the intermolecular interactions in bulk.

3. Conclusion

DFT calculations prove *a close correlation between the molecular conformation of Li_2-DPGO ⇄ DPODO (i.e. closed vs. linear) and the oxidation state*. In the gas-phase, the closed structure is not stable in -2 charge state (i.e. a distorted linear structure is formed); in the bulk structures, while the configuration is still of linear in the presence of two Li atoms, the system is energetically stabilized, pointing out the role of intermolecular interactions as function of the redox state. The DFT results also indicate an energy barrier that can be estimated around 1 eV between reduced and oxidized states. All results are consistent with the model presented and discussed in Figure S29 and experimental observations.

REFERENCES

1. P. Poizot, J. Gaubicher, S. Renault, L. Dubois, Y. Liang, Y. Yao, Opportunities and challenges for organic electrodes in electrochemical energy storage. *Chem. Rev.* **120**, 6490–6557 (2020).

2. B. Esser, F. Dolhem, M. Becuwe, P. Poizot, A. Vlad, D. Brandell, A perspective on organic electrode materials and technologies for next generation batteries. *J. Power Sources* **482**, 228814 (2021).

3. A. E. Lakraychi, F. Dolhem, A. Vlad, M. Becuwe, organic negative electrode materials for metal-ion and molecular-ion batteries: Progress and challenges from a molecular engineering perspective. *Adv. Energy Mater.* **11**, 2101562 (2021).

4. J. Wang, A. Vlad, Empowering *magnesium Energy* **5**, 945–946 (2020).

5. T. B. Schon, B. T. McAllister, P. F. Li, D. S. Seferos, The rise of organic electrode materials for energy storage. *Chem. Soc. Rev.* **45**, 6345–6404 (2016).

6. S. Gottis, A. L. Barres, F. Dolhem, P. Poizot,Voltage gain in lithiated enolate-based organic cathode materials by isomeric effect. *ACS Appl. Mater. Interfaces* **6**, 10870–10876 (2014).

7. H. Jia, T. Quan, X. Liu, L. Bai, J. Wang, F. Boujioui, R. Ye, A. Vlad, Y. Lu, J. F. Gohy, Core-shell nanostructured organic redox polymer cathodes with superior performance. *Nano Energy* **64**, 103949 (2019).

8. A. Jouhara, E. Quarez, F. Dolhem, M. Armand, N. Dupré, P. Poizot, Tuning the chemistry of organonitrogen compounds for promoting all-organic anionic rechargeable batteries. *Angew. Chem. Int. Ed. Engl.* **58**, 15680–15684 (2019).

9. G. Dominique, J.-M. Tarascon, Rocking-chair or lithium-ion rechargeable lithium batteries. *Adv. Mater.* **6**, 408–412 (1994).

10. M. Sid, B. Scrosati, Lithium-ion rechargeable batteries. *J. Power Sources* **51**, 79–104 (1994).

11. A. E. Lakraychi, E. Deunf, K. Fahsi, P. Jimenez, J. P. Bonnet, F. Djedaini-Pilard, M. Bécuwe, P. Poizot, F. Dolhem, An air-stable lithiated cathode material based on a 1,4-benzenedisulfonate backbone for organic Li-ion batteries. *J. Mater. Chem. A* **6**, 19182–19189 (2018).

12. A. Jouhara, N. Dupré, A.C. Gaillot, D. Guyomard, F. Dolhem, P. Poizot, Raising the redox potential in carboxyphenolate-based positive organic materials via cation substitution. *Nat. Commun.* **9**, 4401 (2018).

13. D. Rambabu, A. E. Lakraychi, J. Wang, L. Sieuw, D. Gupta, P. Apostol, G. Chanteux, T. Goossens, K. Robeyns, A. Vlad, An electrically conducting li-ion metal-organic framework. *J. Am. Chem. Soc.* **143**, 11641–11650 (2021).

14. L. Sieuw, A. E. Lakraychi, D. Rambabu, K. Robeyns, A. Jouhara, G. Borodi, C. Morari, P. Poizot, A. Vlad, Through-space charge modulation overriding substituent effect: Rise of the redox potential at 3.35 V in a lithium-phenolate stereoelectronic isomer. *Chem. Mater.* **32**, 9996–10006 (2020).

15. L. Bernard, A. Jouhara, E. Querez, Y. Leviex-Souid, S. L. Caër, P. Tran-Van, S. Renault, P. Poizot, Influence of polymorphism on the electrochemical behavior of dilithium (2,3-dilithium-oxy)-terephthalate vs Li. *Inorganics* **10**, 62 (2022).

16. J. Wang, A.E. Lakraychi, X. Liu, L. Sieuw, C. Morari, P. Poizot, A. Vlad, Conjugated sulfonamides as a class of organic lithium-ion positive electrodes. *Nat. Mater.* **20**, 665–673, (2021).

17. J. Wang, X. Liu, H. Jia, P. Apostol, X. Guo, F. Lucaccioni, X. Zhang, Q. Zhu, C. Morari, J.F. Gohy, A. Vlad, A high-voltage organic framework for high-performance Na- and K-ion batteries. *ACS Energy Lett.* **7**, 668–674 (2022).

18. J. Wang, X. Guo, P. Apostol, X. Liu, K. Robeyns, L. Gence, C. Morari, J. F. Gohy, A. Vlad, High performance Li-, Na-, and K-ion storage in electrically conducting coordination polymers. *Energ. Environ. Sci.* **15**, 3923–3932 (2022).

19. H. Yang, J. Lee, J. Y. Cheong, Y. Wang, G. Duan, H. Hou, S. Jiang, I. D. Kim, Molecular engineering of carbonyl organic electrodes for rechargeable metal-ion batteries: Fundamentals, recent advances, and challenges. *Energ. Environ. Sci.* **14**, 4228–4267 (2021).

20. H. Nishide, T. Suga, Organic Radical Battery. *Electrochem. Soc. Interface* **14**, 32–36 (2005).

21. H. Nishide, K. Oyaizu, Toward Flexible Batteries. *Science* **319**, 737–738 (2008).

22. M. Armand, S. Grugeon, H. Vezin, S. Laruelle, P. Ribière, P. Poizot, J.M. Tarascon, Conjugated dicarboxylate anodes for Li-ion batteries. *Nat. Mater.* **8**, 120–125 (2009).

23. Z. Song, Y. Qian, M.L. Gordin, D. Tang, T. Xu, M. Otani, H. Zhan, H. Zhou, D. Wang, Polyanthraquinone as a reliable organic electrode for stable and fast lithium storage. *Angew. Chem. Int. Ed. Engl.* **54**, 13947–13951 (2015).

24. H. Chen, M. Armand, G. Demailly, F. Dolhem, P. Poizot, J. M. Tarascon, From biomass to a renewable LixC6O6 organic electrode for sustainable Li-ion batteries. *ChemSusChem* **1**, 348–355 (2008).

25. W. Guo, Y. X. Yin, S. Xin, Y. G. Guo, L. J. Wan, Superior radical polymer cathode material with a two-electron process redox reaction promoted by graphene. *Energ. Environ. Sci.* **5**, 5221–5225 (2012).

26. T. Janoschka, M. D. Hager, U. S. Schubert, Powering up the future: Radical polymers for battery applications. *Adv. Mater.* **24**, 6397–6409 (2012).

27. D. Beaudoin, T. Maris, J. D. Wuest, Constructing monocrystalline covalent organic networks by polymerization. *Nat. Chem.* **5**, 830–834 (2013).

28. P. Bibulić, I. Rončević, V. Bermanec, H. Vančik, Polymerization of 1,4-dinitrosobenzene: Kinetics and submicrocrystal structure. *Croat. Chem. Acta* **90**, 1–7 (2017).

29. R. Rathore, J. S. Kim, J. K. Kochi, Catalytic autoxidation of benzoquinone dioximes with nitrogen oxides: Steric effects on the preparation of monomeric dinitrosobenzenes. *J. Chem. Soc. Perkin Trans.* **1**, 2675–2684 (1994).

30. G. G. Kokkinidis, A reaction model for the ECECE mechanism: Reduction of p-benzoquinone dioxime on Pt/M (upd) modified electrodes in $HClO_4$ solutions. *J. Electroanal. Chem. Interfacial Electrochem.* **257**, 239–255 (1988).

31. H. Feuer, *The Chemistry of the Nitro and Nitroso Groups, Part 1*. (Interscience, 1969).

32. B. G. Gowenlock, G. B. Richter-Addo, Dinitroso and polynitroso compounds. *Chem. Soc. Rev.* **34**, 797–809 (2005).

33. J. H. Trepagnier, J. V. Vaughen, N. J. Woodstown, Preparation of dinitrosobenzenes (U.S. Patent No. 2,419,976). United States of American patent (1947).

34. G. Gallo, A. Mihanović, I. Rončević, R. Dinnebier, H. Vančik, Crystal structure and ON-OFF polymerization mechanism of poly(1,4-phenyleneazine-N,N-dioxide), a possible wide bandgap semiconductor. *Polymer* **214**, 123235 (2021).

35. N. P. Hacker, Investigation of the polymerization of 1,4-dinitrosobenzene by low-temperature infrared and UV absorption spectroscopy. *Macromolecules* **26**, 5937–5942 (1993).

36. A. Lorraine, C. Mailer, G. G. Brian, J. M. Lain, Properties and spectroscopic studies of polymeric dinitrosobenzenes. *J. Chem. Soc. Perkin Trans.* **2**, 243–245 (1992).

37. L. Sieuw, A. Jouhara, É. Quarez, C. Auger, J. F. Gohy, P. Poizot, A. Vlad, A H-bond stabilized quinone electrode material for Li-organic batteries: The strength of weak bonds. *Chem. Sci.* **10**, 418–426 (2019).

38. A. K. Sillitoe, M. M. Harding, 3,4-Diphenylfurazan N-oxide. *Acta Crystallogr. B Struct. Cryst. Crystal Chem.* **34**, 2021–2022 (1978).

39. Z.-X. Yu, P. Caramella, K. N. Houk, Dimerizations of nitrile oxides to furoxans are stepwise via dinitrosoalkene diradicals: A density functional theory study. *J. Am. Chem. Soc.* **125**, 15420–15425 (2003).

40. Q. Zhao, Z. Zhu, J. Chen, Molecular engineering with organic carbonyl electrode materials for advanced stationary and redox flow rechargeable batteries. *Adv. Mater.* **29**, 1607007 (2017).

41. B. Huskinson, M. P. Marshak, C. Suh, S. Er, M. R. Gerhardt, C. J. Galvin, X. Chen, A. Aspuru-Guzik, R. G. Gordon, M. J. Aziz, A metal-free organic-inorganic aqueous flow battery. *Nature* **505**, 195–198 (2014).

42. L. Sieuw, B. Ernould, J.F. Gohy, A. Vlad, On the improved electrochemistry of hybrid conducting-redox polymer electrodes. *Sci. Rep.* **7**, 4847 (2017).

43. A. Vlad, K. Arnould, B. Ernould, L. Sieuw, J. Rolland, J. F. Gohy, Exploring the potential of polymer battery cathodes with electrically conductive molecular backbone. *J. Mater. Chem. A* **3**, 11189–11193 (2015).

44. C. Zhao, Z. Chen, W. Wang, P. Xiong, B. Li, M. Li, J. Yang, Y. Xu, In situ electropolymerization enables ultrafast long cycle life and high-voltage organic cathodes for lithium batteries. *Angew. Chem. Int. Ed. Engl.* **59**, 11992–11998 (2020).

45. B. Häupler, A. Wild, U. S. Schubert, Carbonyls: Powerful organic materials for secondary batteries. *Adv. Energy Mater.* **5**, 1402034 (2015).

46. Y. Shi, J. Yang, J. Yang, Z. Wang, Z. Chen, Y. Xu, Quinone-amine polymer nanoparticles prepared through facile precipitation polymerization as ultrafast and ultralong cycle life cathode materials for lithium-ion batteries. *Adv. Funct. Mater.* **32**, 2111307 (2022).

47. L. S. Xie, S. S. Park, M. J. Chmielewski, H. Liu, R. A. Kharod, L. Yang, M. G. Campbell, M. Dincă, Isoreticular linker substitution in conductive metal-organic frameworks with through-space transport pathways. *Angew. Chem. Int. Ed. Engl.* **59**, 19623–19626 (2020).

48. Agilent, CrysAlisPro Version 1.171.37.35, Agilent Technologies UK Ltd, Yarnton, England (2014).

49. G. M. Sheldrick, A short history of SHELX. *Acta Crystallogr. A* **64**, 112–122 (2008).

50. S. Wang, L. Wang, K. Zhang, Z. Zhu, Z. Tao, J. Chen, Organic $Li_4C_8H_2O_6$ nanosheets for lithium-ion batteries. *Nano Lett.* **13**, 4404–4409 (2013).

51. S. Renault, S. Gottis, A.L. Barrès, M. Courty, O. Chauvet, F. Dolhem, P. Poizot, A green Li–organic battery working as a fuel cell in case of emergency. *Energ. Environ. Sci.* **6**, 2124–2133 (2013).

52. V. A. Samsonov, L. V. Volodarskii,G. K. Khisamutdinov, Formation of 1,1,4,4-tetramethoxy-2,3,5,6-tetrahydroximinocyclohexane by the interaction of trinitrosophloroglucinol with hydroxylamine hydrochloride in methanol. *Chem. Heterocycl. Compd.* **33**, 471–474 (1997).

53. J. H. Trepagnier, W., Del., and John V. Vaughen, Woodstown, N. J. Preparation of dinitrosobenzenes (U.S. Patent No. 2,419,976) 1947.

54. A. Altomare, C. Cuocci, C. Giacovazzo, A. Moliterni, R. Rizzi, N. Corriero, A. Falcicchio, EXPO2013: A kit of tools for phasing crystal structures from powder data. *J. Appl. Cryst.* **46**, 1231–1235 (2013).

55. B. Kariuki, International Union of Crystallography – Extended Software/Methods Development Issue.

56. M. W. Schmidt, K. K. Baldridge, J. A. Boatz, S. T. Elbert, M. S. Gordon, J. H. Jensen, S. Koseki, N. Matsunaga, K. A. Nguyen, S. J. Su, T. L. Windus, M. Dupuis, J. A. Montgomery, General atomic and molecular electronic structure system. *J. Comput. Chem.*, **14**, 137–1363 (1993).

57. R. Peverati, D. G. Truhlar, Improving the accuracy of hybrid meta-GGA density functionals by range separation. *J. Phys. Chem. Lett.* **2**, 2810–2817 (2011).

58. P. Ordejón, E. Artacho, J. M. Soler, Self-consistent order-N density-functional calculations for very large systems. *Phys. Rev. B* **53**, R10441-R10444 (1996).

59. J. P. Perdew, A. Ruzsinszky, G. I. Csonka, O. A. Vydrov, G. E. Scuseria, L. A. Constantin, X. Zhou, K. Burke, Restoring the density-gradient expansion for exchange in solids and surfaces. *Phys. Rev. Lett.* **100**, 136406 (2008).

60. J. Quertinmont, A. Carletta, N. A. Tumanov, T. Leyssens, J. Wouters, B. Champagne, Assessing density functional theory approaches for predicting the structure and relative energy of salicylideneaniline molecular switches in the solid state. *J. Phys. Chem. C* **121**, 6898–6908 (2017).

61. S. Grimme, Semiempirical GGA-type density functional constructed with a long-range dispersion correction. *J. Comput. Chem.* **27**, 1787–1799 (2006).
